# Two new siderophores produced by *Pseudomonas* sp. NCIMB 10586: The anti-oomycete non-ribosomal peptide synthetase-dependent mupirochelin and the NRPS-independent triabactin

**DOI:** 10.3389/fmicb.2023.1143861

**Published:** 2023-03-24

**Authors:** Camille Grosse, Nathalie Brandt, Pierre Van Antwerpen, René Wintjens, Sandra Matthijs

**Affiliations:** ^1^Unité de Recherche NaturaMonas, Institut de Recherche LABIRIS, Brussels, Belgium; ^2^RD3 – Pharmacognosy, Bioanalysis and Drug Discovery and Analytical Platform of the Faculty of Pharmacy, Université Libre de Bruxelles, Brussels, Belgium; ^3^Unité Microbiologie, Chimie Bioorganique et Macromoléculaire, Department of Research in Drug Development (RD3), Faculty of Pharmacy, Université Libre de Bruxelles, Brussels, Belgium

**Keywords:** siderophore, *Pseudomonas*, antimicrobial, *Globisporangium*, gene regulation, iron uptake, competition, natural products

## Abstract

**Introduction:**

*Globisporangium ultimum* is an oomycetal pathogen causing damping-off on over 300 different plant hosts. Currently, as for many phytopathogens, its control relies in the use of chemicals with negative impact on health and ecosystems. Therefore, many biocontrol strategies are under investigation to reduce the use of fungicides.

**Results:**

In this study, the soil bacterium *Pseudomonas* sp. NCIMB 10586 demonstrates a strong iron-repressed *in vitro* antagonism against *G. ultimum* MUCL 38045. This antagonism does not depend on the secretion of the broad-range antibiotic mupirocin or of the siderophore pyoverdine by the bacterial strain. The inhibitor molecule was identified as a novel non-ribosomal peptide synthetase (NRPS) siderophore named mupirochelin. Its putative structure bears similarities to other siderophores and bioactive compounds. The transcription of its gene cluster is affected by the biosynthesis of pyoverdine, the major known siderophore of the strain. Besides mupirochelin, we observed the production of a third and novel NRPS-independent siderophore (NIS), here termed triabactin. The iron-responsive transcriptional repression of the two newly identified siderophore gene clusters corroborates their role as iron scavengers. However, their respective contributions to the strain fitness are dissimilar. Bacterial growth in iron-deprived conditions is greatly supported by pyoverdine production and, to a lesser extent, by triabactin. On the contrary, mupirochelin does not contribute to the strain fitness under the studied conditions.

**Conclusion:**

Altogether, we have demonstrated here that besides pyoverdine, *Pseudomonas* sp. NCIMB 10586 produces two newly identified siderophores, namely mupirochelin, a weak siderophore with strong antagonism activity against *G. ultimum,* and the potent siderophore triabactin.

## Introduction

Most oomycetal species of the genus *Globisporangium*, formerly classified in the genus *Pythium* (Uzuhashi et al., 2010), are plant necrotrophic pathogens that infect young juvenile tissue. *G. ultimum* Uzuhashi (Trow), Tojo & Kakish (syn. *Pythium ultimum* Trow, 1901) causes damping-off and root rot on over 300 diverse hosts including fruits, vegetables, fields, ornamentals and forestry crops (Lazreg et al., 2013; Farr and Rossman, 2014; Kamoun et al., 2015). During the infection, mycelia and oospores infect the young feeder roots, and the germinating seeds and seedlings, leading to major losses. On mature plants this disease decreases the root biomass, causing symptoms of nutrient deficiency and thus significant reductions in yield (Schroeder et al., 2013). The most common method to control *Globisporangium* infections is based on a limited number of registered chemicals which overuse threatens human health, causes ecological concerns, and leads to the emergence of resistant isolates (Lamichhane et al., 2017; Pradhan et al., 2017).

To avoid the use of fungicides, one of many biocontrol strategies being investigated for disease management relies on the use of siderophores. These latter are small (<2,000 Da) iron-chelating compounds synthesized by diverse organisms such as plants, bacteria, and fungi in iron-limited conditions (Hider and Kong, 2010; Khan et al., 2018). The scavenging of oxidized iron (Fe^3+^) from the environment and, therefore, the contribution to iron homeostasis in the organism is the major role attributed to these molecules. However, numerous studies have underlined other potential biological functions for these natural compounds, such as transport of other essential metals (zinc, manganese, molybdenum, or vanadium) and protection against copper response during infection or against oxidative stress (Johnstone and Nolan, 2015; Katumba et al., 2022). Furthermore, many research articles report for some bacterial strains a correlation between siderophore production and improvement of their antimicrobial activity, presumably based on iron scavenging competition, but only few siderophores have been identified as true antimicrobials (Kloepper et al., 1980a,b; Buysens et al., 1996; Phoebe et al., 2001; Haas and Défago, 2005; Matthijs et al., 2007; Sayyed and Chincholkar, 2009; Adler et al., 2012; Zeng et al., 2018).

*Pseudomonas* species are Gram-negative bacteria that produce a wide diversity of siderophores. In particular, fluorescent pseudomonads produce the class of fluorescent pyoverdines as major siderophore. To date, about 100 distinct pyoverdines have been described (Hider and Kong, 2010). In addition, other siderophores, generally less complex and with a lower affinity for Fe^3+^ than pyoverdine, are also synthesized by fluorescent and non-fluorescent *Pseudomonas* strains such as enantio/pyochelin, pseudomonine, nocardamin, corrugatin-like siderophores (i.e., corrugatin, ornicorrugatin and histicorrugatin), yersiniabactin, thioquinolobactin, achromobactin and pyridine-2,6-bis(thiocarboxylic acid) (Meyer and Abdallah, 1980; Cornelis and Matthijs, 2002; Matthijs et al., 2007; Schalk et al., 2020). Many pseudomonads are ubiquitous bacteria in agricultural soils. These strains possess numerous attributes suitable for use as biocontrol agents. The ease of maintenance and rapid growth *in vitro* of these strains, combined with their ability to quickly consume seed and root exudates, and their aptitude to colonize both the rhizosphere and the interior of plants by competing aggressively with other microorganisms, make them well suited as biocontrol and growth-promoting agents. Furthermore, they produce a wide spectrum of bioactive metabolites responsible for the suppression of diverse soil pathogens (Weller, 2007).

*Pseudomonas* sp. NCIMB 10586 well-known to yield the clinically important antibiotic mupirocin which has a broad-spectrum activity against both Gram-positive, and to a lesser extent, Gram-negative bacteria (Sutherland et al., 1985) is also known to produce the pyoverdine PYO_13525_ (Matthijs et al., 2014). In this study, we show that the strain displays a strong iron-repressed *in vitro* antagonism against the oomycete *G. ultimum* MUCL 38045, suggesting the involvement of a siderophore.

The aim of this study is to identify the growth inhibitory molecule and to characterize its biosynthetic gene cluster. We found that the antagonistic molecule is a novel non-ribosomal peptide synthetase (NRPS) siderophore, designated mupirochelin. Its predicted structure shares resemblance with several siderophores, especially with the putative structures of coelibactin (Bentley et al., 2002) and equibactin (Heather et al., 2008), as well as with the bioactive compounds transvalencin A (Hoshino et al., 2004) and uniformides A and B (Hara et al., 2022). Besides pyoverdine and mupirochelin, the present study identifies a third siderophore produced by NCIMB 10586, which has been denoted triabactin. Assessment of the involvement of each siderophore in the fitness of the strain suggests that mupirochelin is a weak siderophore with great anti-*Globisporangium* activity.

## Materials and methods

### Strains and growth conditions

The strains and plasmids used in this study are shown in Table 1 and Supplementary Table S1. *Pseudomonas* strains were routinely grown at 30°C in casamino acids medium (CAA) (5 g L^−1^ Casamino acids, 0.9 g L^−1^ K_2_HPO_4_, 0.25 g L^−1^ MgSO_4_.7H_2_O) (Cornelis et al., 1992), in an in-house medium 853 (Matthijs et al., 2013) or in succinate minimal medium (Anjaiah et al., 1998). *Escherichia coli* strains were grown in 853 at 37°C. *Alcanivorax dieselolei* DSM 16502 was grown in tryptic soy broth (TSB) medium (Titan Biotech Ltd.) or CAA. *Luteibacter anthropi* DSM 23190 was grown in 2216 marine broth (Millipore). For iron restricted growth, culture media were treated with the iron chelator 2,2′-bipyridine, at concentrations of 40, 60, 80, or 100 μM. When necessary, media were supplemented with ampicillin, chloramphenicol, kanamycin, or tetracycline at a concentration of 100, 25, 50, and 60 μg mL^−1^, respectively. *Globisporangium ultimum* MUCL 38045 was maintained on potatoes dextrose agar (PDA) (Difco) and was grown on GCA (CAA + 0.2% glucose) for the *in vitro* antagonism assay.

**Table 1:** Strains used in this study.

Strains	Relevant genotype/characteristics	Reference
*Pseudomonas* sp. NCIMB 10586	Wild-type, mupirocin producing strain isolated from soil, produces pyoverdine PYO_13525_, mupirochelin and triabactin	Fuller et al. (1971)
*Pseudomonas* sp. NCIMB 10586 deletion mutants:		
10586ΔAT2	Mupirocin-negative AT2 deletion mutant	El-Sayed et al. (2003)
10586ΔpvdL	Pyoverdine-negative *pvdL* deletion mutant, produces mupirochelin and triabactin	This study
10586ΔmchAB	Mupirochelin-negative *mchAB* deletion mutant, produces pyoverdine and triabactin	This study
10586ΔtrbABC	Triabactin-negative *trbABC* deletion mutant, produces pyoverdine and mupirochelin	This study
10586ΔpvdLΔmchAB	Pyoverdine/mupirochelin-negative deletion mutant, produces triabactin	This study
10586ΔpvdLΔtrbABC	Pyoverdine/triabactin-negative deletion mutant, produces mupirochelin	This study
10586ΔmchABΔtrbABC	Mupirochelin/triabactin-negative deletion mutant, produces pyoverdine	This study
10586ΔpvdLΔmchABΔtrbABC	Siderophore-negative mutant	This study
10586ΔpvdLΔtrbABCΔmchQ	Mutant in the *mchQ* TonB-dependent receptor gene in a pyoverdine/triabactin-negative background, produces mupirochelin	This study
10586ΔpvdLΔtrbABCΔmchS	Mutant in the *mchS* TonB-dependent receptor gene in a pyoverdine/triabactin-negative background, produces mupirochelin	This study
10586ΔpvdLΔtrbABCΔmchQΔmchS	Mutant in both *mchQ and mchS* genes in a pyoverdine/triabactin-negative background, produces mupirochelin	This study
10586ΔpvdLΔmchABΔtrbABCΔmchQ	Mutant in *mchQ* TonB-dependent receptor gene in a siderophore-negative background	This study
10586ΔpvdLΔmchABΔtrbABCΔmchS	Mutant in *mchS* TonB-dependent receptor gene in a siderophore-negative background	This study
10586ΔpvdLΔmchR	Deletion mutant in the *mchR* transcriptional regulator coding gene in a pyoverdine-negative background, produces triabactin	This study
10586ΔpvdLΔmchAB::trbA	Mini-Tn5 mutant with a Tn5 insertion in *trbA* gene, Tc^R^	This study
*E. coli* MC1061	Cloning strain	
*E. coli* HB101/pME497	Triparental mating helper strain	
*E. coli* SM10 (λpir)/pTnModO-Tc	Plasposon host for bi-parental mating	
*Alcanivorax dieselolei* DSM 16502	Wild-type	DSMZ
*Luteibacter anthropi* DSM 23190	Wild-type	DSMZ
*Globisporangium ultimum* MUCL 38045	*G. ultimum* (Trow) Uzuhashi et al., Syn. *Pythium ultimum* Trow (previously *Pythium debaryanum*)	BCCM/ MUCL

### *In vitro* growth inhibition assay against *Globisporangium ultimum* MUCL 38045

The *G. ultimum* MUCL 38045 growth inhibition activity was evaluated by an *in vitro* growth inhibition assay as described in Matthijs et al. (2007). Briefly, growth inhibition was assessed on GCA and GCA supplemented with 100 μM FeCl_3_ by measuring the radius of the no growth zone of the phytopathogen around the *Pseudomonas* colony. All tests were performed three times with three replicates.

To assess the anti-*Globisporangium* activity of mupirochelin, a 5 mm plug of MUCL 38045 was placed at the center of a GCA plate. After 24 h of incubation, four filter-paper disks were placed at approximately 2.7 cm from the center of the plate. Ten μL of semi-purified mupirochelin, prepared as described later, was used to impregnate each paper disk and a control was made with pure DMSO.

### Screening of a transposon insertion library for an antagonism-negative mutant of *Pseudomonas* sp. NCIMB 10586

To identify the genes involved in the *in vitro* antagonism of *Pseudomonas* sp. NCIMB 10586 against *G. ultimum* MUCL 38045, a Tn*5* mutagenesis was carried out through bi-parental mating of NCIMB 10586 with *E. coli* carrying the plasposon pTnModO-Tc (Table 1). Briefly, mid-log phase cultures of *E. coli* SM10 (λpir), the host of the plasposon, was mixed with NCIMB 10586 in a 1:1 ratio. Strain NCIMB 10586 was kept at 37°C for 1 h just before mixing both strains to inactivate its restriction-modification system. Transposon insertions in NCIMB 10586 were selected on CAA supplemented with 60 μg mL^−1^ tetracycline and 25 μg mL^−1^ chloramphenicol. Subsequently, a bank of 960 transconjugants was screened for mutants showing loss of the ability to inhibit the growth of *G. ultimum* MUCL 38045. Therefore, Tn5 mutants were grown overnight at 30°C in CAA medium in microtiter plates. Three μL of each candidate was inoculated on a GCA plate, in total eight Tn*5* mutants were inoculated equidistant in a circle at 2.7 mm from the centre of the plate. After incubation overnight at 30°C, a 5 mm agar plug with *G. ultimum* mycelium was placed at the centre and the plate was incubated for 2 more days at 30°C. Mutants that showed loss of growth inhibition were selected and further confirmed by the Petri plate assay. Four antagonism-negative mutants were obtained and characterized molecularly as described in Matthijs et al. (2009).

### Screening of a transposon insertion library for a siderophore-negative mutant of 10586∆pvdL∆mchAB

To identify a putative third siderophore system a Tn*5* mutagenesis was carried out on the pyoverdine and mupirochelin-negative mutant 10586∆pvdL∆mchAB (Table 1) with the plasposon pTnModO-Tc as described above. A bank of 3,425 transconjugants was screened for mutants showing no chelating activity when streaked on chrome-azurol S (CAS) agar plates. One candidate, 10586ΔpvdLΔmchAB::trbA (Table 1), was obtained and characterized molecularly as described in Matthijs et al. (2009).

### *In silico* analyses

*In silico* analyses of the mupirochelin gene cluster were conducted using the bioinformatics software tools antiSMASH 6.0 (Blin et al., 2019), SBSPKS v3 (Agrawal and Mohanty, 2021), PKS/NRPS Analysis (Bachmann and Ravel, 2009) and PRISM 3 (Skinnider et al., 2017; Table 2). The predicted function for each open reading frame (ORF) was assigned by comparing the translated product with known proteins in public databases (Supplementary Table S2).

**Table 2:** Comparison of substrate prediction for the NRPS enzymes of the *mch* gene cluster by four different software.

Software	MchA substrate prediction	MchB substrate prediction
antiSMASH v6.0.1	cysteine – cysteine	cysteine
PRISM 3	cysteine – cysteine	cysteine
SBSPKS v3	threonine – cysteine	cysteine
PKS/NRPS Analysis	threonine – cysteine	cysteine

### Construction of in-frame deletion mutants in *Pseudomonas* sp. NCIMB 10586

In-frame deletion mutants were constructed in the three siderophore systems. To be able to work in a pyoverdine and triabactin-negative background the pyoverdine biosynthetic gene *pvdL* coding for the chromophore (∆pvdL) and the complete triabactin gene cluster (∆trbABC) were deleted in NCIMB 10586. In order to study the mupirochelin siderophore gene cluster, both mupirochelin biosynthetic NRPS genes *mchA* and *mchB* (∆mchAB), each TonB-dependent receptor gene namely *mchQ* (∆mchQ) and *mchS* (∆mchS) or/and the transcriptional regulator gene *mchR* (∆mchR) were deleted. The method used is the same as described in Matthijs et al. (2016). For the deletion of the NRPS genes *pvdL* and *mchAB,* internal 5′ and 3′ fragments of the genes to be deleted were amplified and cloned in pUK21. For the other genes to be deleted the 5′ and 3′ flanking regions were amplified and cloned in pUK21. The list of the primers used to amplify the fragments is given in Supplementary Table S3, the constructed plasmids are given in Supplementary Table S1. The obtained mutations were confirmed by PCR amplification and subsequent sequencing of the fragments (using verification primers, Supplementary Table S3). Pyoverdine and mupirochelin productions were verified by LC–MS and CAS assay.

### Evaluation methods of siderophore production and growth under iron-limiting conditions

Siderophore production was detected by means of the colorimetric chrome-azurol S assay (CAS) (Schwyn and Neilands, 1993). Therefore, 5 μL of an overnight culture grown in CAA adjusted to OD_600_ = 0.5 was inoculated on CAS agar and kept at 30°C. After 24/48 h of incubation the area of the orange diffusion zone around the colony was measured with ImageJ software (Schneider et al., 2012) by subtracting the area of the colony to the total orange surface. Growth curves were obtained using a PowerWave™ XS2 microplate spectrophotometer with Gen5™ software (BioTek Instuments, Inc., Winooski, VT, USA). Overnight cultures in CAA were adjusted to OD_600_ = 0.02, subjected to a 10-fold dilution and 5 μL was inoculated in a 96-wells microplate containing 195 μL CAA medium per well. 2,2′-bipyridine concentrations were used at 40, 60, and 80 μM. To prevent condensation the microplate covers were coated with 10 mL 0.05% Triton X-100 in 20% ethanol and air-dried (Brewster, 2003). The plate was placed in the microplate reader for incubation at 30°C for 48 h with a high shaking speed. Absorbance readings at 600 nm were taken every hour. All experiments were performed three times with two replicates each time.

### Reverse transcription qPCR (RT-qPCR)

Bacterial cultures were grown overnight in CAA medium. Twenty mL of OD_600_ = 0.2 of the overnight culture was used to inoculate 180 mL CAA before incubation at 30°C with shaking at 200 rpm. When the culture reached OD_600_ = 0.2 the sample was divided into six 30 mL subcultures, each one was supplemented with FeCl_3_ (Merck) to a final concentration of 0, 0.05, 0.1, 0.2, 0.3, or 0.5 μM and the amended subcultures were further incubated for 1 h at 30°C with shaking at 200 rpm. Cells were then pelleted, washed, and resuspended in fresh CAA medium, and RNA was protected with the addition of RNAprotect Bacteria Reagent (Qiagen). Total RNA was extracted with RNeasy Mini kit (Qiagen) and treated with RNase free DNA Set (Qiagen) to remove any genomic DNA contamination. RNA integrity was analyzed by agarose gel electrophoresis, and its purity and concentration were calculated using a NanoDrop DeNovix DS-11 spectrophotometer (DeNovix Inc., Wilmington, DE, USA). Reverse transcription was conducted on 1 μg of total RNA with RevertAid H Minus First Strand cDNA synthesis kit with oligo(dt) primers (Thermo Scientific), in accordance with manufacturer’s instructions. Prior to RT-qPCR assays, cDNA was purified with QIAquick PCR Purification kit (Qiagen). Quantification was performed using Fast Start Essential DNA Green Master (Roche Applied Science) and conducted on a LightCycler 96 (Roche) with the appropriate primers (Supplementary Table S4) designed using the software AmplifX 2 (by Nicolas Jullien; Aix-Marseille Univ, CNRS, INP, Inst Neurophysiopathol, Marseille, France^1^). For normalization, selection of a reference gene was made with RefFinder online tool (Xie et al., 2012) among six candidates *algD* (GDP-mannose 6-dehydrogenase), *fabD* (malonyl CoA-acyl carrier protein transacylase), *gyrA* (DNA gyrase subunit A), *oprL* (peptidoglycan-associated protein), *rho* (transcription termination factor Rho) and *rpsL* (30S ribosomal protein S12) (Supplementary Table S5). Results were obtained from at least three replicates and presented as the expression of a given gene relative to that of *rho*. The same approach was used by supplementing the subcultures with ZnCl_2_ (Fisher Chemical) or NiCl_2_ (Fluka) to a final concentration of 0, 2, 4, 6, 8, or 10 μM.

For comparison of gene expression between the exponential and stationary growth phases, an overnight preculture was used to inoculate two cultures of 30 mL CAA. Cells were harvested at OD_600_ = 0.3 for the exponential phase and after 17 h 30 of incubation (OD_600_ = ± 0.6) for the stationary phase. Total RNA extraction and RT-qPCR assays were then conducted as described earlier. For normalization, selection of a reference gene was made with RefFinder online tool among six candidates *algD*, *gyrA*, *nadB* (L-aspartate oxidase), *oprL*, *pks* (polyketide synthase) and *rpsL* (Supplementary Table S6). Results were presented as the expression of a given gene relative to the *nadB* reference gene.

To investigate the involvement of the membrane receptors MchQ and MchS in mupirochelin uptake by the strain, the effect of mupirochelin on the expression of both receptor coding genes was studied. Therefore, ±18 h old filter-sterilized supernatants (OD_600_ = 0.4) of a mupirochelin producing (NCIMB∆pvdL∆trbABC) and non-producing mutant (NCIMB∆pvdL∆mchAB∆trbABC) (Table 1) were added to cultures of strains of which one or both receptor genes, *mchQ* and *mchS,* were deleted in a siderophore-negative background. To this end, 6 mL OD_600_ = 0.2 from the overnight precultures of the mutants NCIMB∆pvdL∆mchAB∆trbABC, NCIMB∆pvdL∆mchAB∆trbABC∆mchQ and NCIMB∆pvdL∆mchAB∆trbABC∆mchS (Table 1) were used to inoculate 60 mL CAA. The cultures were incubated at 30°C with shaking at 200 rpm. When OD_600_ of the cultures reached 0.2, the sample was divided into two 23 mL subcultures, each one was supplemented with 7 mL of siderophore-negative or mupirochelin-positive filter-sterilized supernatant. The amended subcultures were further incubated for 1 h at 30°C with shaking at 200 rpm and RNA was extracted as described above. Results were obtained from three replicates and presented as the expression of a given gene relative to that of *rho*.

### Analysis of mupirochelin production and metal complexation by LC–MS

Strain NCIMB 10586 and its mutants (Table 1) were grown in succinate minimal medium using the medium scale method as described in Matthijs et al. (2009). LC–MS analyses were performed using an Alliance e2695 separation module equipped with a 2998 PDA detector (Waters, Milford, MA, USA) and coupled to an Altus SQ mass detector (Perkin Elmer) (ES ionization, positive mode, cone voltage 49 V, capillary voltage 3.1 kV, source temperature 150°C, desolvation temperature 600°C). The used column was a C18 Alltima (Grace) (250 × 4.6 mm, 10 μm) with a flow rate of 0.5 mL min^−1^ and a gradient from H_2_O/CH_3_OH 9:1 containing 0.1% formic acid to H_2_O/CH_3_OH 1:9 containing 0.1% formic acid. Mass spectral data were collected and analyzed using Empower 3 software. Mupirochelin peaks were identified using the mupirochelin-negative mutant as reference.

To determine if *A. dieselolei* DSM 16502 and *L. anthropi* DSM 23190 (Table 1) can produce mupirochelin, the strains were grown in, respectively, CAA and 2216 marine broth supplemented with 100 μM 2,2′-bipyridine. Siderophores were isolated from their supernatants and analyzed by LC–MS as described earlier.

For mupirochelin-metal complexes identification, 25 μM FeCl_3_, GaNO_3_, NiCl_2_ or ZnCl_2_ was added to the semi-purified supernatants of the mupirochelin producer 10586ΔpvdLΔtrbABC, and the siderophore negative mutant 10586ΔpvdLΔmchABΔtrbABC before LC–MS analysis. Controls were realized without addition of metal.

### Mupirochelin characterization by MS/MS

MS/MS analysis of mupirochelin was carried out with a LC-QTOF6520 (Agilent Technologies, Palo alto, CA, USA). Briefly, the chromatographic method used the previous Alltima C18 column at 25°C with 0.025% TFA and 0.075% formic acid in water (solvent A) and CH_3_CN (solvent B) in the following gradient: 0 min A 100%; 3 min A 90%; 33 min A 30%; 40 min A 30%; 41 min A 100%. The column was equilibrated during 20 min between each acquisition. The source was set at 325°C with drying gas at 11 L min^−1^, nebulizer 55 psig, capillary voltage at 4000 V and fragmentor at 125 V. The MS range acquisition was between 100 and 1,000 *m*/*z*. The MS/MS acquisition between 50 and 1,000 *m*/*z* was performed in targeted MS/MS as the corresponding mass of 509.1345 *m*/*z* was detected around 28 min. Accordingly, the target MS approach was applied between 18 and 38 min with CE at 3, 5, 10, 20, 30, 50 eV of energy. Five μL of each sample were injected in both MS and MS/MS. The data were analyzed thanks to MassHunter Qualitative Analysis (Agilent Technologies, Palo alto, CA, USA). The fragments identification was based on the accumulation of the MS/MS spectra at the different collision energy.

### Large scale mupirochelin purification for growth inhibition assay

Mupirochelin was purified from 6 × 1 L of 48 h old culture supernatant of 10586ΔpvdLΔtrbABCΔmchQ grown at 26°C in CAA medium. Bacterial cells were removed by centrifugation at 6,728 g, during 30 min and the supernatant was divided into three 2 L samples. One sample served as control without addition of any metal, and 1 mM FeCl_3_ or 1 mM GaNO_3_ was added to the two other culture supernatants. After a 30 min incubation the supernatants were filtered on Whatman No. 113V paper and passed on C18 column activated with methanol and rinsed with H_2_O and 5% CH_3_CN. The semi-purified siderophore-metal complex was eluted with 70% CH_3_CN, evaporated using compressed air and lyophilized. After lyophilization the semi-purified samples were resuspended in 500 μL DMSO.

### Large scale mupirochelin purification for structure determination

Mupirochelin was purified from 25 × 1 L of 48 h old culture supernatant of NCIMBΔpvdLΔtrbABCΔmchQ grown at 26°C in CAA medium. If needed, GaNO_3_ was added to a final concentration of 1 mM. The cell-free supernatant was prepared by centrifugation at 6,728 g, for 30 min and was filtered on Whatman No. 113V paper. The resulting sample was passed on C18 column activated with methanol and rinsed with H_2_O and 5% CH_3_CN. The semi-purified siderophore was eluted with 70% acetonitrile, evaporated using compressed air and lyophilized. Further purification was done by semi-preparative HPLC on a SunFire Prep C18 column (100 Å, 5 μM particle size, 19 mm × 250 mm) with a flow rate of 20 mL min^−1^ and a gradient going from H_2_O/CH_3_CN 1:4 to H_2_O/CH_3_CN 1:2.5. UV detection was performed at 254 nm. The fractions obtained were directly analyzed by LC–MS as described above and the purified mupirochelin was further lyophilized for concentration and storage.

### Phylogenetic analysis of *Pseudomonas* strains containing the mupirochelin gene cluster or uptake genes in their genome

To study the phylogenetic distribution of *Pseudomonas* strains containing the mupirochelin gene cluster or uptake genes in their genome, the DDBJ/EMBL/GenBank databases were searched through BLAST queries for strains having the mupirochelin gene cluster in their genome and for strains having only the TonB-dependent receptors MchQ or MchS. When a *Pseudomonas* strain identified by BLAST search as containing a putative mupirochelin gene cluster was present in our in-house *Pseudomonas* collection, its supernatant was semi-purified using the medium-scale method and analyzed by LC–MS for the presence of mupirochelin in the sample (Supplementary Table S7). The ability to inhibit the growth of *G. ultimum* of putative mupirochelin producer strains was tested using the *in vitro* antagonism assay.

The *rpoD* gene sequence of the strains with a mupirochelin gene cluster or uptake genes, and of the *Pseudomonas* type strains was downloaded from GenBank and trimmed (Mulet et al., 2011; Girard et al., 2021). Phylogenetic analysis based on partial *rpoD* gene sequences was performed using CLUSTALX and MEGA v11.0.11 (Larkin et al., 2007; Tamura et al., 2021). The maximum-likelihood method was used with the Tamura-Nei model and the topological robustness was evaluated by bootstrap analysis based on 1,000 replicates.

### Statistical analysis

The statistical tests used are specified in the figure legends wherever appropriate. GraphPad Prism 6 was used for statistical analyses.

## Results

### Iron-regulated anti-*Globisporangium* activity is linked to siderophore production

**Figure 1:**
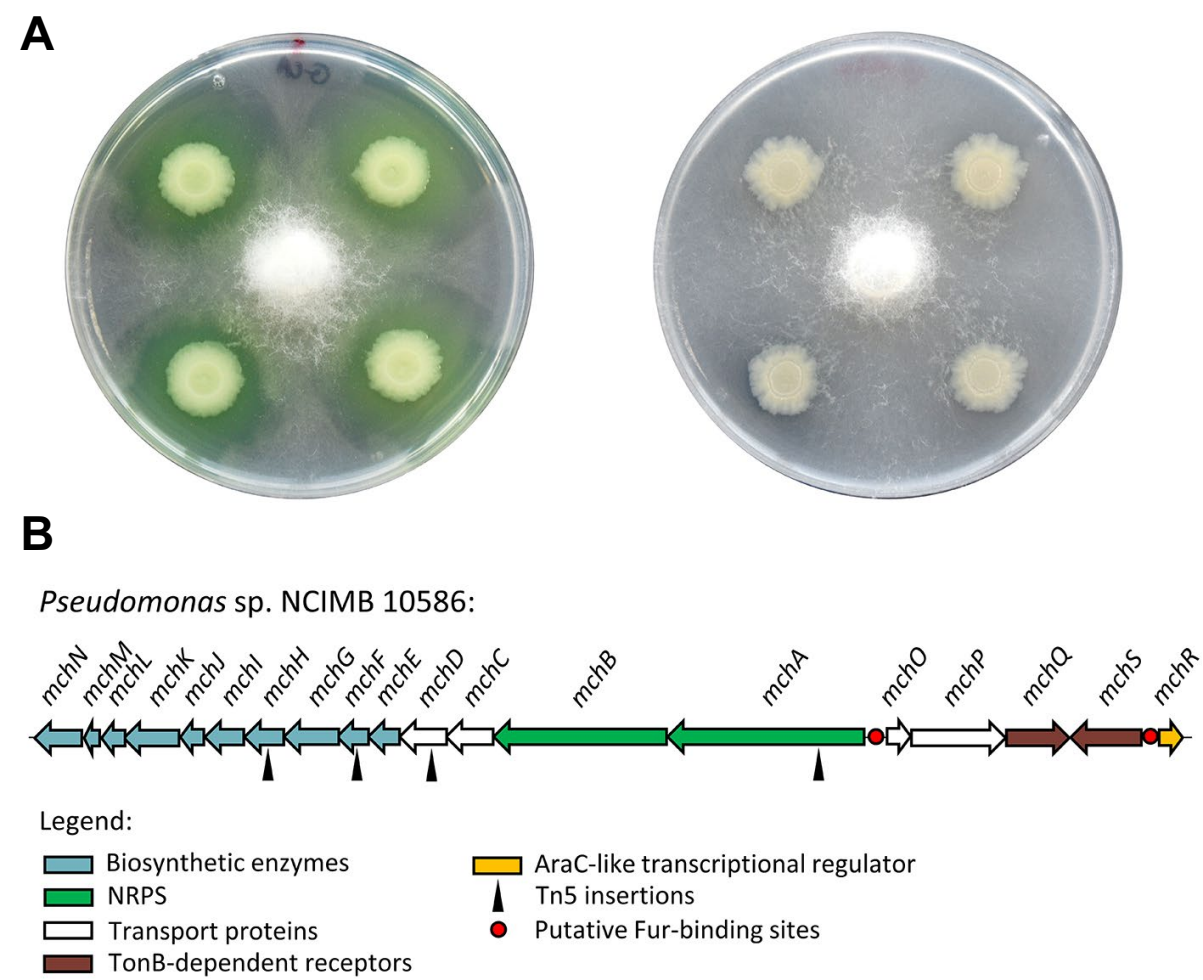
*Pseudomonas* sp. NCIMB 10586 growth inhibitory activity is related to iron availability. **(A)** Results of the antagonism assay of the ∆AT2 mutant against *G. ultimum* MUCL 38045 in iron-limited (left) and iron-rich (right) medium. The mupirocin-negative mutant is still able to inhibit the growth of the phytopathogen. However, addition of 100 μM FeCl_3_ in the medium is responsible for the loss of the antagonism activity. **(B)** Organization of the gene cluster for the synthesis of mupirochelin in *Pseudomonas* sp. NCIMB 10586. Transposon insertions are indicated by black triangles. Red circles represent hypothetical Fur-binding sites. Predicted genes functions obtained by BLASTp and InterPro analyses: *mchN*: isochorismate synthase, *mchM*: isochorismate-pyruvate lyase, *mchL*: thioesterase, *mchK*: (2,3-dihydroxybenzoyl) adenylate synthase, *mchJ*: vicinal oxygen chelate (VOC) family protein, *mchI*: thiazolinyl imide reductase, *mchH*: cytochrome P450 protein, *mchG*-*mchF*: class I SAM-dependent methyltransferases, *mchE*: saccharopine dehydrogenase, *mchD*-*mchC*: ABC transporter ATP-binding proteins, *mchA*-*mchB*: NRPS synthetases, *mchO*: ABC-type multidrug transport system, ATPase component, *mchP*: membrane protein, *mchQ-mchS*: TonB-dependent receptors, *mchR*: AraC family transcriptional regulator.

*Pseudomonas* sp. NCIMB 10586 inhibits the growth of *G. ultimum* MUCL 38045 under iron-restricted conditions (Figure 1; Supplementary Figure S1A). Since the *in vitro* antagonism of the mupirocin-negative mutant 10586∆AT2 (El-Sayed et al., 2003) was not affected (Figure 1A), a role of the antibiotic in the observed growth inhibition is excluded. To identify the nature of the compound responsible for this anti-oomycete activity, a Tn*5* mutagenesis was carried out on the wild type, and the transconjugants were screened for a complete loss of the anti-*Globisporangium* activity. Four antagonism-negative candidates were obtained, and sequencing of their Tn*5* flanking regions revealed their insertions in a 36 kb-long gene cluster of 19 open reading frames (ORFs) coding for a putative siderophore, here named mupirochelin (Figure 1B).

The mupirochelin (*mch*) gene cluster contains two divergent operons and two genes, *mchS* and *mchR,* presumed to be involved in membrane transport and in transcriptional regulation, respectively. The first operon *mchABCDEFGHIJKLMN*, in which the four Tn5 insertions were mapped (Figure 1B), is predicted to be responsible for the biosynthesis of mupirochelin. The operon *mchOPQ* and *mchS* are predicted to be involved in mupirochelin internalization into the bacterial cell, and *mchR* is coding for a putative AraC-like transcriptional regulator of the *mch* gene cluster (Supplementary Table S2). Iron concentration may also regulate its transcription through the ferric uptake regulator (Fur) pathway as putative Fur boxes were found in both promoter regions, and considering this universal regulator is commonly related to siderophore biosynthesis (Supplementary Table S8; Hunt et al., 1994; Reimmann et al., 1998; Li et al., 2019).

**Figure 2:**
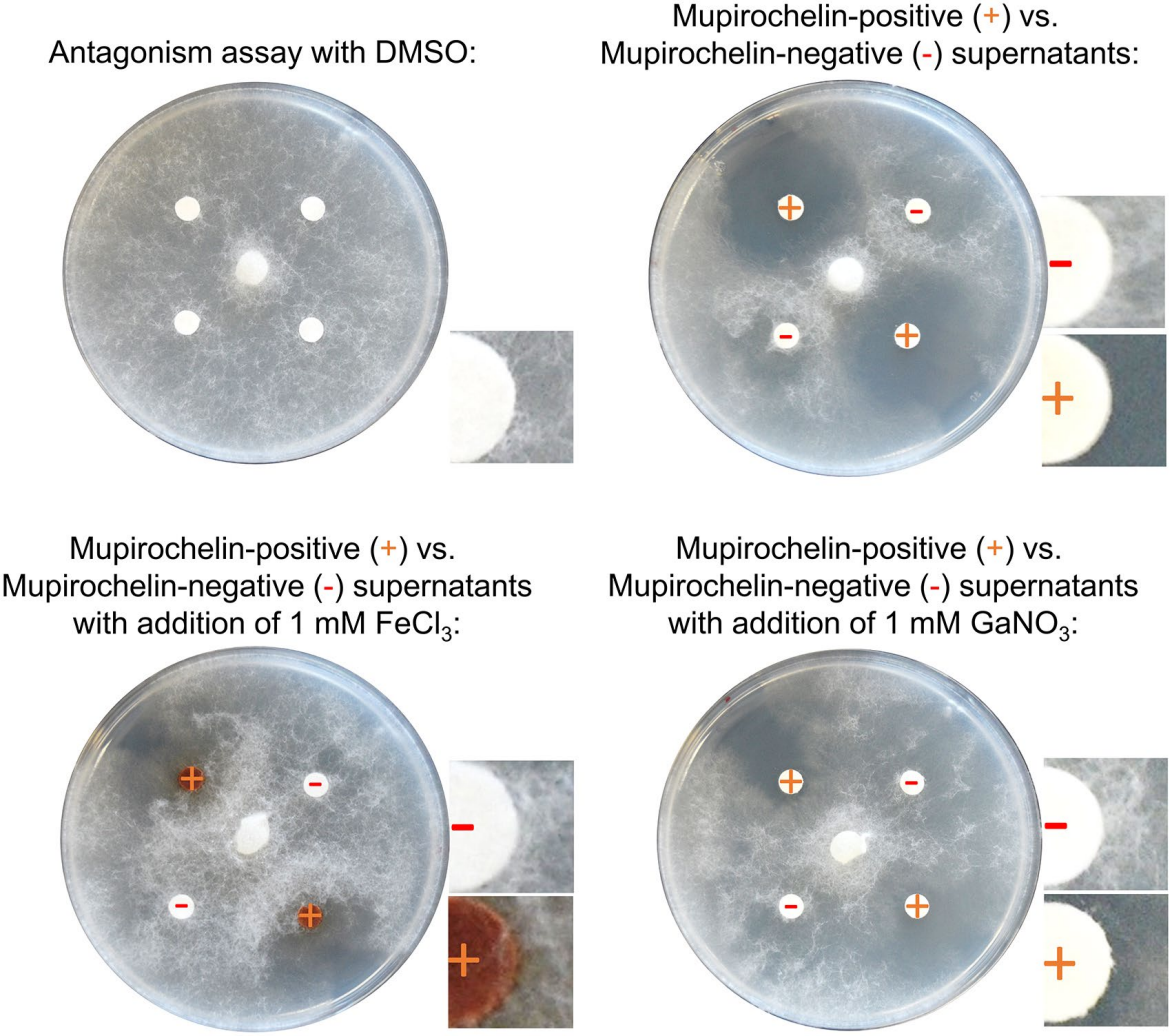
Growth inhibitory assays of mupirochelin and its chelated forms with iron and gallium. The growth inhibitory assay is realized with the semi-purified supernatants of 10586∆pvdL∆trbABC∆mchQ (mupirochelin-positive) and 10586∆pvdL∆mchAB∆trbABC (mupirochelin-negative) mutants. Samples were prepared with or without the addition of 1 mM FeCl_3_ or GaNO_3_ and resuspended in DMSO. On the upper-left corner, the control shows that DMSO does not present a growth inhibitory activity against *G. ultimum*. *Apo*-mupirochelin is necessary for the inhibition of the oomycete expansion (upper-right corner). However, ferri-mupirochelin has lost most of its activity. On the lower-left panel, the growth of the phytopathogen is slowed down. *G. ultimum* hyphae are able to progress on the ferri-mupirochelin impregnated disks. The same observation is made for the gallium-mupirochelin complex.

To further investigate the role of mupirochelin in the anti-*Globisporangium* activity, the semi-purified siderophore and its complexes with Fe^3+^ or Ga^3+^ were used for *in vitro* growth inhibition assay (Figure 2). Only *apo-*mupirochelin was able to clearly inhibit the growth of the oomycete, both Fe^3+^-mupirochelin and Ga^3+^-mupirochelin complexes lost their antagonism activity. However, the progression of the phytopathogen was slowed down by these two semi-purified complexes, possibly due to the presence of a small fraction of *apo*-mupirochelin.

### Predictive structure of mupirochelin indicates a mixed-type siderophore

**Figure 3:**
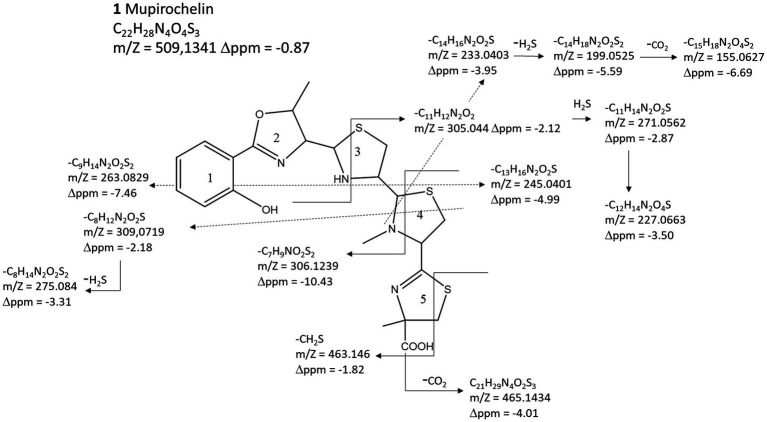
Structure prediction of mupirochelin. Predicted chemical structure of mupirochelin based on its MS/MS fragmentation pattern. Each arrow represents the cleavage of a particular bond.

Comparison of the LC–MS injection of mupirochelin-positive and mupirochelin-negative semi-purified supernatants provides an extracted chromatogram with 5 different peaks at 509.1345 (see Supplementary Figure S2) which are postulated to be 5 isomers of the same structure. Consequently, a target MS/MS acquisition was endorsed with a selected precursor at 509.1345 and between 18 and 38 min. The MS/MS spectra of each peak give the same fragmentation pattern confirming the presence of isomers. Therefore, the MS/MS pattern of the third peak at 28.3 min was used to predict the putative structure. The fragments provide a putative structure of mupirochelin (**1**) with a total elemental composition of C_22_H_28_N_4_O_4_S_3_ (Figure 3). The MS/MS fragmentation followed the classical fragmentation with mainly breakage in alpha of heteroatoms (N and S) as illustrated by the fragmentation pattern of the putative homologs, pyochelin, yersiniabactin and escherichelin (Moree et al., 2012; Schulz et al., 2020). Indeed, we observed the neutral loss of CO_2_ (C_21_H_29_N_4_O_2_S_3_), the fragmentation of cycle 5, 3 and 4 followed by the neutral loss of H_2_S and CO_2_ but also the fragmentation between cycle 3 and 4 (Figure 3). The final predicted structure shares similarities with several siderophores, such as the predicted aryloxazoline siderophore coelibactin (**4**) (Bentley et al., 2002), the arylthiazoline siderophores yersiniabactin (**2**) (Drechsel et al., 1995), pyochelin (**5**) (Cox et al., 1981), thiazostatin (**6**) (Shindo et al., 1989), escherichelin (**7**) (Ohlemacher et al., 2017), ulbactin F and G (**8**) (Igarashi et al., 2016), and pre-equibactin (**3**) (Heather et al., 2008). Furthermore, structural similarities are also found with the bioactive compounds transvalencin A (**9**) (Hoshino et al., 2004) and uniformides A and B (**10–11**) (Hara et al., 2022; Supplementary Figure S3).

It is noteworthy that the fragmentation of cycle 4 into fragments at 233.0403 *m*/*z* and 306.1239 *m*/*z* is in favor of a N-methyl derivative on this cycle. This type of methylation is also found in compounds **6**, **9**, **10** and **11**. Further analysis of mupirochelin structure have proven unsuccessful as the pure siderophore is unstable in its *apo* form and, remained weakly soluble and unstable in the solvents tested (ethyl acetate, methanol, acetonitrile, DMSO) when complexed with Ga^3+^.

### Prediction of mupirochelin biosynthetic pathway

**Figure 4:**
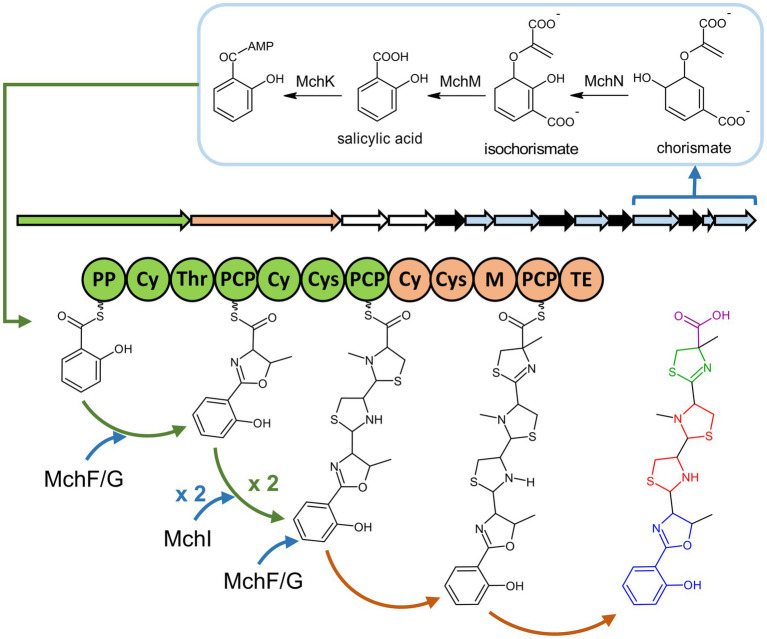
Predicted biosynthesis pathway of the mupirochelin siderophore. The first step is the conversion of chorismate to salicylic acid by MchN and MchM enzymes. The compound is then activated by MchK and transferred to the phosphopantheteine-binding domain (PP) of the NRPS enzyme MchA. The first adenylation domain (Thr) is predicted to activate a threonine, which is then condensed, cyclized by the heterocyclization domain (Cy) and transferred to the next peptide carrier protein domain (PCP). N-methylation of the oxazoline ring is predicted to be induced by one of the two SAM-methyltransferase enzymes MchF or MchG. Coupling of a cysteine unit by the second adenylation domain of MchA (Cys) and reduction of the new thiazoline ring formed by the putative thiazolinyl imide reductase MchI are predicted to be iteratively repeated twice. The second SAM-methyltransferase (MchF or MchG) is predicted to be responsible for the methylation of the third unit thiazolidine ring. The final cysteine unit is coupled by the second NRPS enzyme MchB which possesses a methylation domain (M) predicted to realize a C-methylation before release of the compound by the final thioestherase domain (TE). Operon coloring scheme: *mchA*: green, *mchB*: orange, transport-related genes: white, function unknown in the biosynthesis pathway: black, additional biosynthetic genes: blue. Mupirochelin coloring scheme: aryloxazoline group: blue, thiazolidine rings: red, thiazoline ring: green, carboxyl group: purple.

In the *mch* gene cluster the two non-ribosomal peptide synthetases (NRPS), designated MchA and MchB, are predicted to assemble a putative 3-residue backbone consisting out of Cys/Thr – Cys – Cys (Table 2). A thioesterase (TE) domain, which catalyzes the release of the NRPS product, was found in MchB (Figure 4).

Surprisingly, these predictions obtained from the assembly line differ from the predicted 4-residue structure given by the MS/MS analysis. Exceptions in the NRPS co-linearity rule are not uncommon in literature as various types of iterative operations are observed (Mootz et al., 2002). Based on these predictions, the MS/MS analysis presented above, and the biosynthetic pathways of pyochelin (**5**) and yersiniabactin (**2**) (Crosa and Walsh, 2002; Schalk et al., 2020), we propose a putative biosynthetic pathway for mupirochelin (Figure 4). At the beginning of the assembly line, MchN and MchM are responsible for the conversion of chorismate to salicylic acid. Then, salicylic acid is activated by MchK and the resulting salicyl-AMP is transferred to the first NRPS enzyme MchA. The first amino acid unit of the siderophore structure is activated by the first adenylation domain of MchA and is transferred to the following peptide carrier protein domain (PCP). Based on the MS/MS analysis, we hypothesized that this first substrate of MchA is a threonine, as proposed for other siderophores (Heather et al., 2008; Yan et al., 2019; Shyam et al., 2021). Coupling of the amino acid to the salicylate is performed by successive steps of condensation, cyclization and dehydration. The oxazoline ring is probably N-methylated by one of the putative SAM-methyltransferases MchF or MchG during the biosynthetic process. Iteratively, two cysteine units are added and cyclized by MchA, and both thiazoline rings are probably reduced by the reductase MchI based on the MS/MS analysis. The thiazolidine ring of the second cysteine unit could be methylated by either MchF or MchG. The second NRPS enzyme MchB catalyzes the addition, the cyclization and the methylation of the last cysteine unit before release of the siderophore through hydrolyzation by the final thioesterase domain of the assembly line. MchL thioesterase is predicted to remove wrongly charged molecules from the peptidyl carrier protein of MchA and MchB enzymes, as it is the case for its homologue PchC found in the pyochelin gene cluster (Reimmann et al., 2004). The function of MchH, MchJ, and MchL in the biosynthesis of mupirochelin remains currently unknown.

### Phylogenetic distribution of the mupirochelin biosynthesis and uptake genes in the *Pseudomonas* genus

To investigate whether mupirochelin production is widespread or limited to a few species, BlastX searches in the DDBJ/EML/Genbank databases using both receptors *mchQ* and *mchS* were performed. For candidates with a (almost) complete *mch* gene cluster or with the operons involved in transport and regulation alone, and for the *Pseudomonas* type strains, the *rpoD* gene was downloaded and trimmed (accessed on October 17, 2022). A maximum likelihood tree (TN + G + I model) was then constructed (Supplementary Figure S4). This tree contains 114 strains, including NCIMB 10586, all identified as bearing in their genome a gene cluster predicted to be involved in the biosynthesis and the uptake of mupirochelin. For a handful of these strains present in our in-house collection, the iron-repressed growth inhibitory activity against *G. ultimum* was observed and the production of mupirochelin was confirmed by mass analysis (Supplementary Table S7; Supplementary Figure S4).

The ability to produce the siderophore mupirochelin is limited to strains of the *P. fluorescens* lineage (Supplementary Figure S4). Most of the candidates are found in the *P. fluorescens* and the *P. corrugata* subgroup. Strain NCIMB 10586 belongs to the *P. fluorescens* subgroup. The type strain of *P. baetica* (*P. koreensis* subgroup), *P. helleri* (*P. fragi* subgroup), *P. fulva* (*P. putida* group) and a few closely related strains to these type strains also have a putative *mch* gene cluster in their genome. The *mch* gene cluster is also found in a strain closely related to the phytopathogen *P. syringae* pv. *syringae* (Supplementary Figure S4).

Not all the strains have the same organization of the mupirochelin gene cluster as *Pseudomonas* sp. NCIMB 10586. Clusters of strains are found whereby one gene of the *mch* gene cluster is not present in their genome. For instance, all the strains of the *P. corrugata* subgroup miss *mchH*, the cytochrome P450 coding gene. Nevertheless, we showed by LC–MS analysis that the type strain of *P. thivervalensis* (*P. corrugata* subgroup) produces mupirochelin thereby demonstrating that the gene is not essential for the biosynthesis of the siderophore. In addition, *Pseudomonas* sp. Xaverov 259 and *Pseudomonas* sp. KBS0710, each isolated from a different biological origin but grouped together (Supplementary Figure S4), both miss the isochorismate-pyruvate lyase gene. It is currently not known whether these strains are able to produce mupirochelin as these strains are not currently in our possession. Finally, a small cluster of three strains in the *P. putida* group, namely *P. fulva* 20-MO00615-0 and 20-MO00618-0, and *P. putida* PSB00048, lack the *mchS* gene coding for a TonB-dependent receptor (Supplementary Figure S4).

The *Pseudomonas* strains most likely acquired and transferred the mupirochelin gene cluster through horizontal gene transfer (HGT). It was observed for *Pseudomonas* sp. 2822–17 and *P. poae* CAP-2018 (Supplementary Figure S4) that their mupirochelin gene cluster is surrounded upstream and downstream by conjugative elements. A highly similar gene cluster was also identified in the genomes of two distant bacterial strains, *A. dieselolei* DSM 16502 and *L. anthropi* DSM 23190 (Supplementary Figure S5). For *A. dieselolei* DSM 16502, the genes of the TonB-dependent receptor *mchS* and of the regulator *mchR* are differently located in the gene cluster. For *L. anthropi* DSM 23190, the latter two genes are missing. Nevertheless, no production of mupirochelin has been detected in the supernatant of both bacterial strains in the conditions studied.

Interestingly, remnants of HGT were found in a group of 10 strains where a transposase is inserted in the intergenic region, between the genes encoding the TonB-dependent receptors MchQ and MchS (Supplementary Figure S4). Remarkably, all the strains that produce or are predicted to produce the siderophore mupirochelin, are likewise foreseen to synthesize the high affinity siderophore pyoverdine as evidenced by the presence in their genome of the *pvdL* gene, encoding the pyoverdine chromophore, and putative pyoverdine gene clusters. The sole exception was the strain *P. fluorescens* ISL-23 for which a *pvdL* gene was not found in its genome. The main siderophore of ISL-23 seems to be a corrugatin-like siderophore (Risse et al., 1998; Matthijs et al., 2008, 2016). Therefore, bacteria producing mupirochelin must produce another high affinity siderophore to be able to capture iron efficiently, suggesting that mupirochelin is a rather weak siderophore.

*Pseudomonas* strains often have a multitude of TonB-dependent siderophore receptors in their genome allowing them to use several exogenous siderophores. Many strains (22) of the *P. gessardii* subgroup, and *Pseudomonas* sp. KBW05 (*P. fluorescens* subgroup), contain a gene cluster for the uptake of mupirochelin (Supplementary Figure S4, indicated in blue) whereby the genes involved in transport and regulation are present (i.e., *mchMN*, *mchOPQ*, *mchS* and *mchR*), but none of the biosynthetic genes. Intriguingly, both TonB-dependent receptor coding genes are present in this small gene cluster involved in the uptake of mupirochelin, which suggests that both MchQ and MchS have a biological role for these strains.

### Identification of a third siderophore system termed triabactin

**Figure 5:**
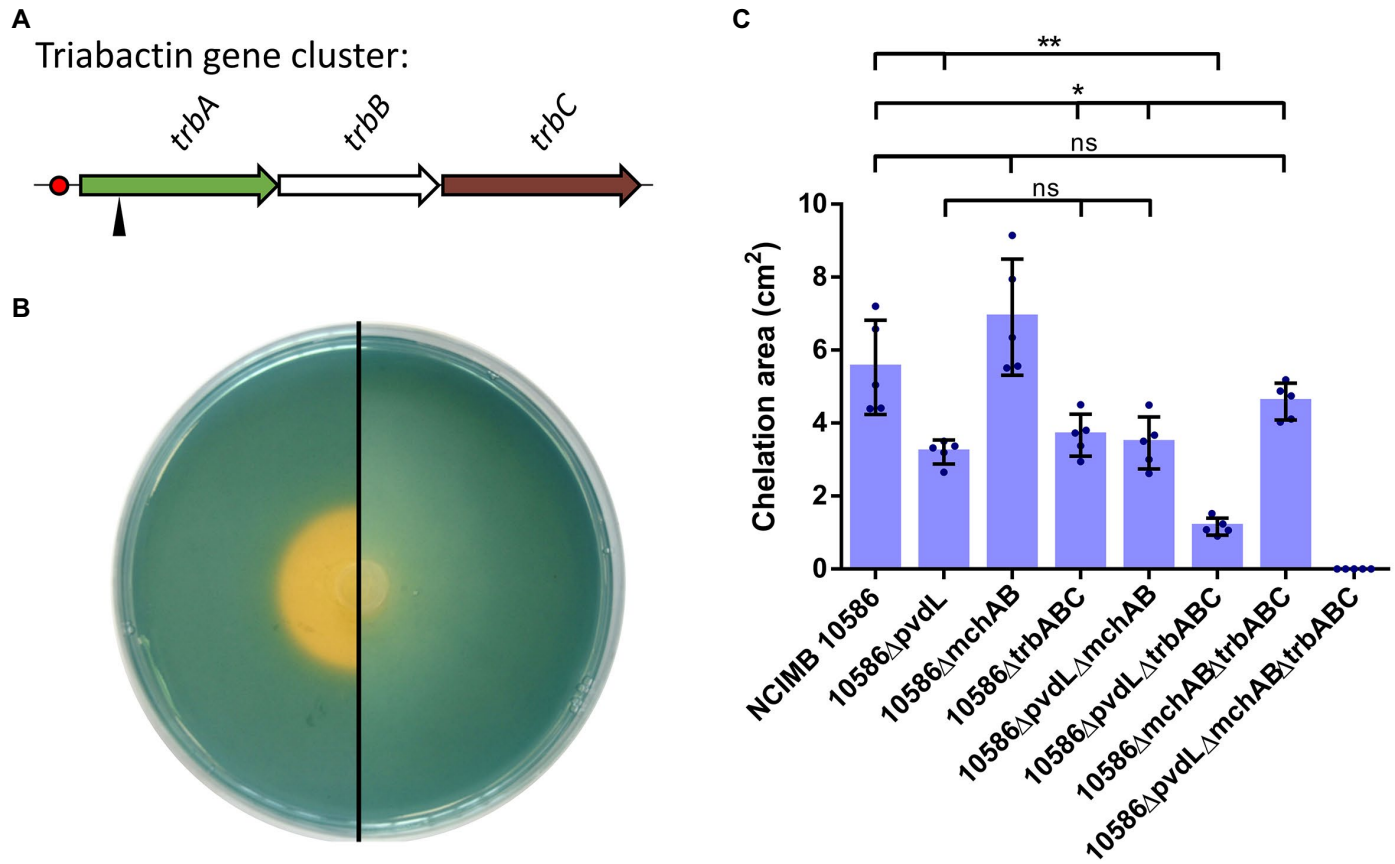
A third siderophore named triabactin is produced by *Pseudomonas* sp. NCIMB 10586. **(A)** Organization of the *trb* gene cluster responsible for triabactin biosynthesis and utilization by the strain. *trbA:* NRPS-independent synthetase, *trbB:* MFS transporter, *trbC:* TonB-dependent receptor. The transposon insertion is indicated by a black triangle. Hypothetical Fur-binding site is represented by a red circle. **(B)** Comparison of the CAS results for the pyoverdine-mupirochelin-negative mutant 10586∆pvdL∆mchAB (left) and the pyoverdine-mupirochelin-triabactin-negative mutant 10586∆pvdL∆mchAB∆trbABC (right). Negative result for 10586∆pvdL∆mchAB∆trbABC strain suggests that the mutant is siderophore-negative. **(C)** Comparison of the chelation areas observed for each siderophore mutant after 48 h incubation on CAS plates. Inactivation of the production of the three siderophores in the strain NCIMB 10586 leads to the absence of iron chelating activity by the mutant. All dots represent biological replicates (*n* = 5). Results of Mann–Whitney *U*-test for between-group pairwise comparison ns *p* > 0.05, *p < 0.05, **p < 0.01, ***p < 0.001.

*Pseudomonas* sp. NCIMB 10586 is already known to produce the siderophore pyoverdine PYO_13525_ (Matthijs et al., 2014). To further characterize the mupirochelin gene cluster, the biosynthetic genes *mchA* and *mchB* were deleted in a pyoverdine-negative background (10586ΔpvdLΔmchAB) (Table 1). The mutant was confirmed by LC–MS for loss of mupirochelin production and its anti-*Globisporangium* activity was assessed by means of the *in vitro* growth inhibition assay. As expected, loss of mupirochelin biosynthesis resulted in loss of growth inhibition against the phytopathogen (Supplementary Figure S1A). Siderophore secretion by this mutant was tested on CAS medium (Figure 5B; Supplementary Figure S1B). Interestingly, the CAS assay did not reveal a significant difference between the pyoverdine-negative mutant 10586ΔpvdL and the double pyoverdine/mupirochelin-negative mutant 10586ΔpvdLΔmchAB, as both show a clear halo around the colony (Figure 5C). This result suggests the production of a third siderophore by the strain. To confirm this hypothesis, a Tn*5* mutagenesis was carried out on the 10586ΔpvdLΔmchAB mutant, and the bank obtained was screened for CAS-negative transconjugants. Molecular characterization of one CAS-negative candidate, mutant 10586ΔpvdLΔmchAB::trbA (Table 1), shows that the Tn5 insertion is in the first gene of a small operon coding for a predicted NRPS-independent siderophore (NIS) which was designated triabactin (Figure 5A; Carroll and Moore, 2018). The triabactin (*trb*) operon consists of three genes which are predicted to code for a putative NRPS-independent siderophore synthase (*trbA*), an MFS transporter (*trbB*) and a TonB-dependent receptor (*trbC*) by means of BlastX analysis. To confirm the identified operon, the entire triabactin gene cluster was deleted in the strain 10586ΔpvdLΔmchAB (Table 1), and the CAS assay was repeated (Figure 5B). The strain 10586ΔpvdLΔmchABΔtrbABC shows a complete loss of siderophore production (Figure 5C) and is therefore referred to as the siderophore-negative mutant.

### Mupirochelin has low CAS chelation activity

To compare all possible combinations of siderophore production on CAS medium for the three siderophore systems, pyoverdine PYO_13525_, mupirochelin and triabactin, additional mutants were constructed (Table 1) and they were all analyzed by the CAS assay (Figure 5C; Supplementary Figure S1B). The results of the mutants that produce only one siderophore show that pyoverdine (mutant 10586ΔmchABΔtrbABC) has the largest CAS chelation activity, followed by triabactin (mutant 10586ΔpvdLΔmchAB). Mupirochelin (mutant 10586ΔpvdLΔtrbABC) has a significantly lower CAS chelation activity than pyoverdine and triabactin (Figure 5C).

In line with these results, it is observed that inactivation of pyoverdine biosynthesis in the wild type (mutant 10586ΔpvdL) is responsible for a decrease of about 42% of the area of chelation around the colony (Figure 5C). Overall, deletion of *pvdL* in all mutants is responsible for a significative decrease in their area of chelation (Figure 5C). Similarly, loss of triabactin production in all the mutants leads to a reduction of their halo on CAS (Figure 5C).

In contrast, suppression of mupirochelin biosynthesis in the wild type (mutant 10586ΔmchAB) and in the pyoverdine-negative mutant (mutant 10586ΔpvdLΔmchAB) does not influence the size of the area of chelation around the colony. Besides, the same deletion introduced in the triabactin-negative mutant (mutant 10586ΔmchABΔtrbABC) leads to a significative increase in the size of the halo. Therefore, the small increase in the CAS halo in mutant 10586ΔmchABΔtrbABC is probably due to an increase in pyoverdine production.

### Growth in iron-limiting conditions imposed by the iron chelator 2,2′-bipyridine

**Figure 6:**
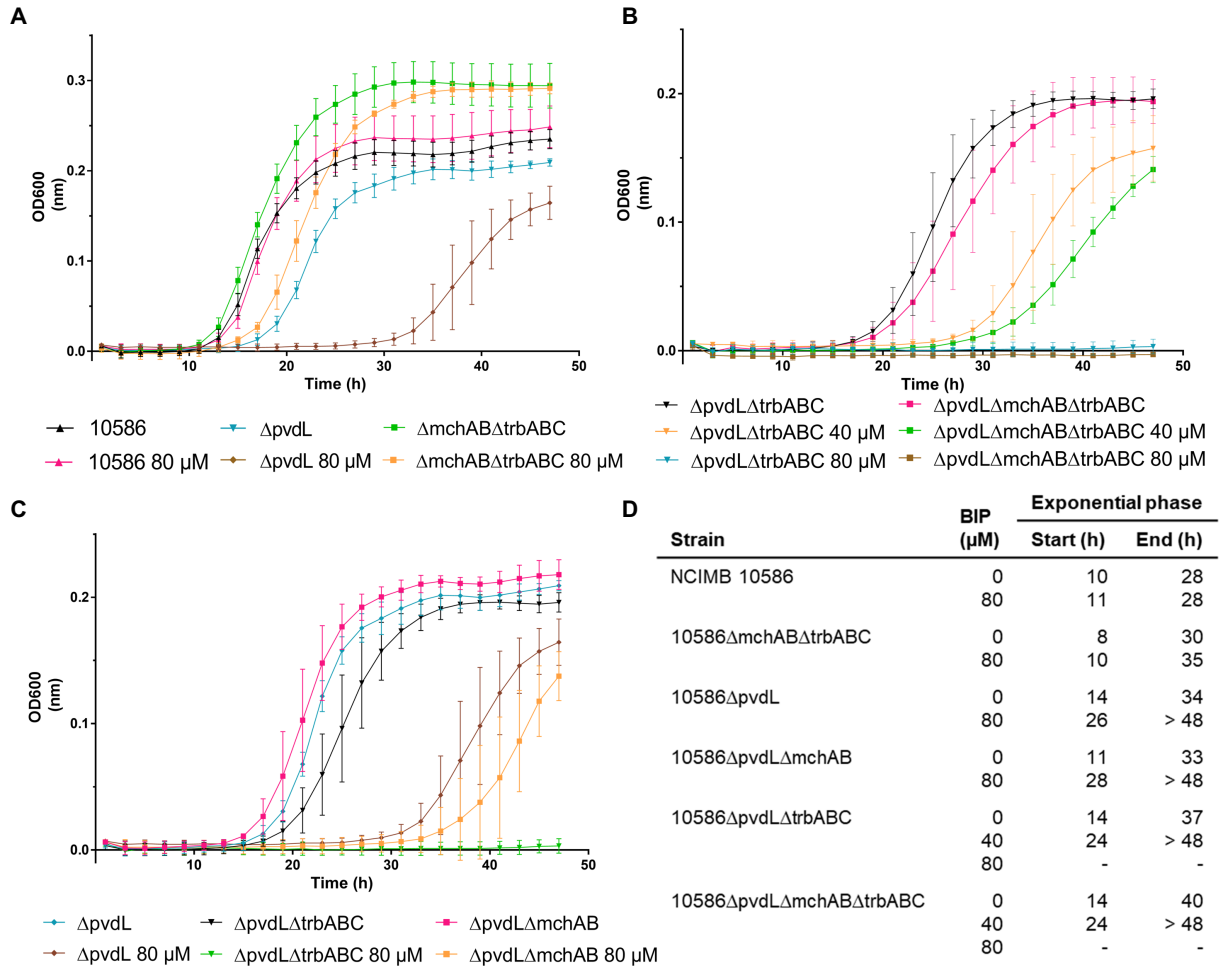
Characterization of the importance of pyoverdine, mupirochelin and triabactin for the growth of the bacterial strain in iron-stress conditions. **(A)** Effect of pyoverdine production on the strain fitness. The 2,2′-bipyridine concentration does not affect the wild type growth curve. 10586∆mchAB∆trbABC mutant which only produces the siderophore pyoverdine is tolerant to the highest 2,2′-bipyridine concentration added, with only a small delay compared to its growth in CAA medium. Surprisingly, the exponential phase of this mutant last longer compared to the wild type, leading to a higher final cell concentration in the culture even under iron limitation. Absence of pyoverdine production has a great impact on 10586∆pvdL growth and reduces its tolerance to 2,2′-bipyridine without erasing it. **(B)** Effect of mupirochelin on the strain fitness. Mupirochelin production by 10586∆pvdL∆trbABC is not sufficient to improve the strain growth compared to the siderophore-negative mutant 10586∆pvdL∆mchAB∆trbABC. Both mutants are unable to grow in 80 μM 2,2′-bipyridine. **(C)** Effect of triabactin on the strain fitness. Triabactin production alone is responsible for the growth up to 80 μM 2,2′-bipyridine. **(D)** Summary of the growth curves obtained for the strains tested. Exponential phase start and end times were determined graphically. Addition of 80 μM 2,2′-bipyridine (BIP) inhibits 10586∆pvdL∆trbABC and 10586∆pvdL∆mchAB∆trbABC growth.

To estimate the contribution of each siderophore to the strain fitness in iron-limited conditions, growth curves were made with increasing concentrations of the iron chelator 2,2′-bipyridine in the culture medium (Figure 6). For the wild type, the similar growth curves observed in the different 2,2′-bipyridine concentrations (0, 40 and 80 μM) indicate that the ability to produce three different siderophores confers to the strain a great tolerance to low iron stress (Figure 6A).

Pyoverdine greatly contributes to the strain growth in iron-restricted conditions. Its absence in mutant 10586∆pvdL results in a longer lag phase and a greater susceptibility for lack of iron availability. Preservation of pyoverdine biosynthesis in mutant 10586∆mchAB∆trbABC is responsible for the recovery of the growth curve profile of the wild type in absence of 2,2′-bipyridine and enables the growth in the highest 2,2′-bipyridine concentration with only a slight delay. These results emphasize pyoverdine importance for the strain. In particular, the mutant that only produces pyoverdine (10586∆mchAB∆trbABC) has a higher final OD than the wild type strain.

In comparison, in CAA medium without 2,2′-bipyridine the siderophore-negative mutant (10586∆pvdL∆mchAB∆trbABC) has a longer lag phase (Figure 6C). This inhibition of siderophore production results in greater susceptibility for 2,2′-bipyridine concentration in the culture medium with a total growth inhibition at 80 μM 2,2′-bipyridine. The mupirochelin producing mutant 10586∆pvdL∆trbABC and the siderophore-negative mutant show similar behaviors. This phenomenon corroborates that mupirochelin is a rather weak siderophore which probably has a weak contribution to the strain fitness in conditions of iron stress (Figure 6B). Finally, triabactin biosynthesis by 10586ΔpvdLΔmchAB enables the mutant to grow in the highest 2,2′-bipyridine concentration (Figure 6C), unlike mupirochelin production which is unable to support the growth of 10586ΔpvdLΔtrbABC.

Under the studied conditions, triabactin appears as an intermediate siderophore between pyoverdine and mupirochelin: it is less supportive of growth in iron restricted conditions imposed by 2,2′-bipyridine than pyoverdine, but it is a stronger siderophore than mupirochelin. These results are in line as those obtained above for the CAS assay.

### MchR is an activator of mupirochelin production

**Figure 7:**
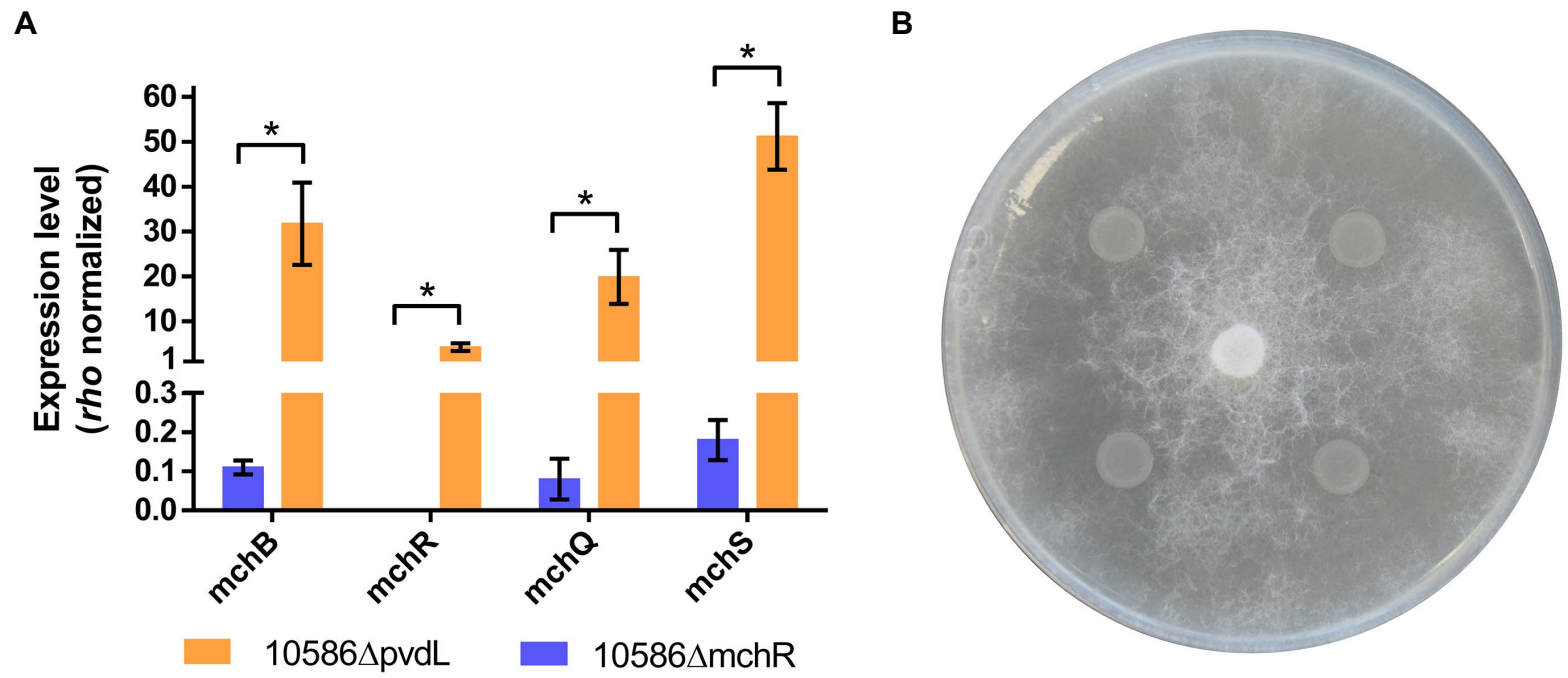
MchR is a transcriptional activator of the *mch* gene cluster. **(A)** Effect of *mchR* deletion on the levels of gene expression for mupirochelin biosynthesis gene *mchB* and both TonB-dependent receptors coding genes *mchQ* and *mchS* in the 10586∆pvdL mutant in exponential growth phase (*n* = 3). Compared to the 10586∆pvdL mutant, absence of MchR biosynthesis in 10586∆pvdL∆mchR leads to a strict repression of the *mch* gene cluster. Results of unpaired Student’s t-test with Welsh’s correction ns *p* > 0.05, *p < 0.05, **p < 0.01, ***p < 0.001. **(B)** Antagonism assay of 10586ΔpvdLΔmchR against *G. ultimum* MUCL 38045. As expected, deletion of *mchR* is responsible for the loss of the strain anti-*Globisporangium* activity.

To determine whether MchR is a repressor or an activator of mupirochelin biosynthesis, *mchR* was deleted in strain 10586ΔpvdL, and mupirochelin production was analyzed by LC–MS and *in vitro* growth inhibition test. The mutant 10586ΔpvdLΔmchR lost mupirochelin production and is unable to inhibit the growth of *G. ultimum* indicating that MchR is an activator (Figure 7B). Transcription analyses of 10586ΔpvdL and 10586ΔpvdLΔmchR mutants by RT-qPCR assay show that the deletion of *mchR* is responsible for the repression of the *mch* gene cluster (Figure 7A). These results confirm that MchR is a transcriptional activator of the *mch* gene cluster.

### MchQ is involved in mupirochelin uptake by *Pseudomonas* sp. NCIMB 10586

**Figure 8:**
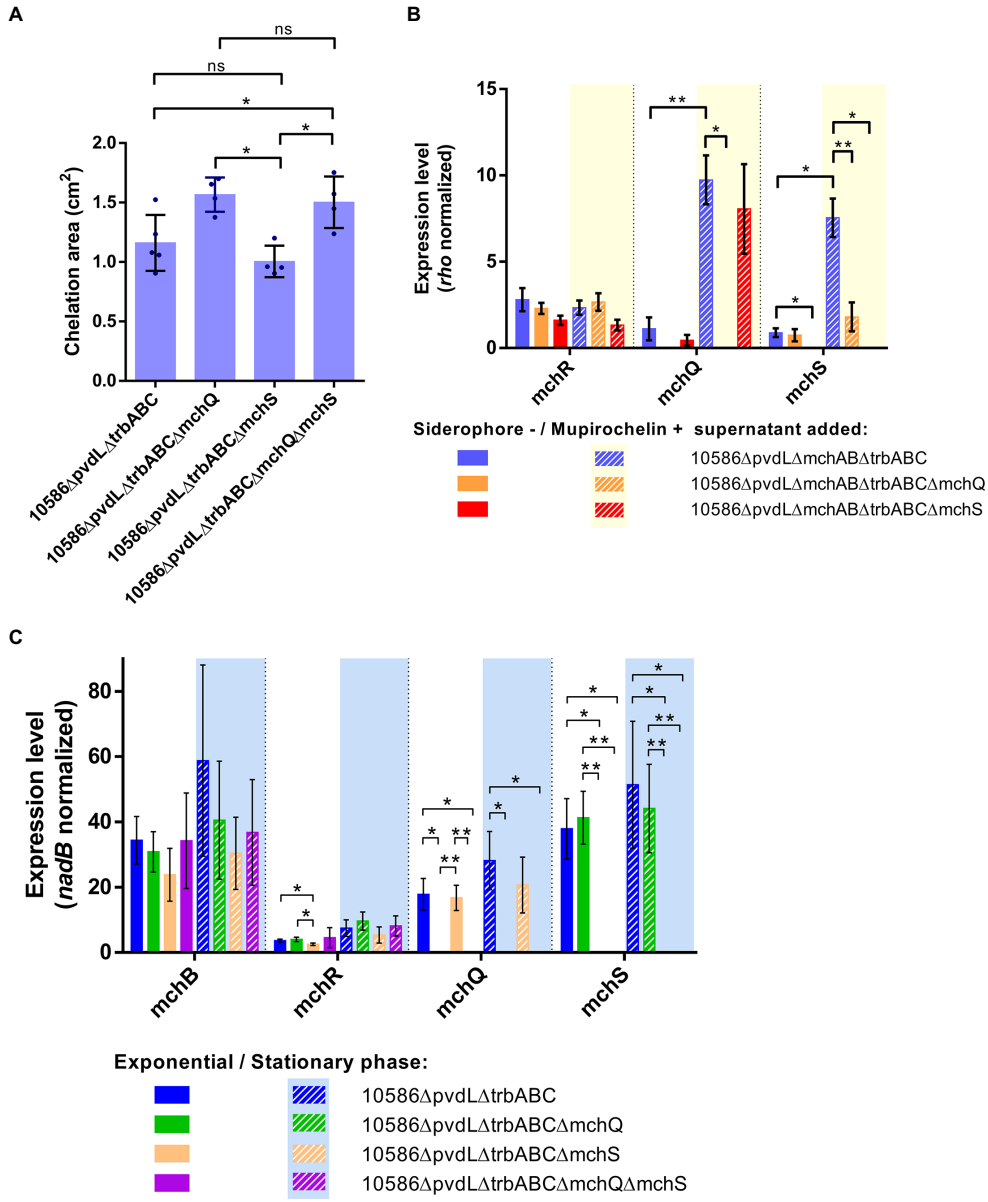
Comparison of MchQ and MchS TonB-dependent membrane receptors involvement in mupirochelin transport and biosynthesis. **(A)** Results of CAS assay for mutants in a pyoverdine and triabactin negative background with additional deletion in *mchQ* or *mchS* genes. Deletion of *mchS* in 10586∆pvdL∆trbABC∆mchS does not affect the area of chelation produced in the CAS medium. On the contrary both 10586∆pvdL∆trbABC∆mchQ and 10586∆pvdL∆trbABC∆mchQ∆mchS mutants show a larger surface of chelation in CAS medium compared with 10586∆pvdL∆trbABC and 10586∆pvdL∆trbABC∆mchS, suggesting that *mchQ* plays a role in mupirochelin uptake by the strain. All dots represent biological replicates (*n* = 4). Results of Mann–Whitney *U*-test for between-group pairwise comparison: ns *p* > 0.05, *p < 0.05, **p < 0.01, ***p < 0.001. **(B)** Expression levels of *mchR, mchQ* and *mchS* genes in siderophore-negative strains incubated with siderophore-negative or mupirochelin-positive supernatants (*n* = 3). *mchR* expression level is not influenced by the addition of mupirochelin in the culture medium. In the strains expressing *mchQ,* the transcription level of the TonB-dependent receptor coding genes is increased by mupirochelin supplementation. The specific deletion of *mchS* does not influence the expression level of the other studied genes. However, the expression level of *mchS* is not affected by the addition of mupirochelin in the culture medium when *mchQ* is deleted, suggesting that MchQ plays a role in mupirochelin uptake. Results of unpaired Student’s t-test with Welsh’s correction *p < 0.05, **p < 0.01, ***p < 0.001. **(C)** Influence of *mchQ* and *mchS* deletions on *mch* transcription. Expression levels of *mchB, mchQ* and *mchS* do not differ between each mutant 10586∆pvdL∆trbABC, 10586∆pvdL∆trbABC∆mchQ, 10586∆pvdL∆trbABC∆mchS, 10586∆pvdL∆trbABC∆mchQ∆mchS (*n* ≥ 3). Results of unpaired Student’s t-test with Welsh’s correction *p < 0.05, **p < 0.01, ***p < 0.001.

Two TonB-dependent receptors are found in the *mch* gene cluster: MchQ and MchS (55% identity at amino acid level) (Figure 1B). The deletion of *mchS* in 10586∆pvdL∆trbABC producing only mupirochelin does not affect the area of its chelation zone around the colony in CAS medium (Figure 8A). In contrast, deletion of *mchQ* in 10586∆pvdL∆trbABC and in 10586∆pvdL∆trbABC∆mchS leads to a significative enlargement of their halo in CAS medium (Figure 8A). These results suggest the involvement of MchQ in mupirochelin uptake by the strain. Loss of the MchQ TonB-dependent receptor, and thereby the entrance of the siderophore into the cell, probably leads to its accumulation around the colony resulting in an increase of the chelation halo area in the CAS assay.

To further investigate the role of the MchQ membrane receptor in mupirochelin uptake, RT-qPCR experiments were conducted on the single and double receptor mutants in a mupirochelin producing strain (pyoverdine/triabactin-negative) in both exponential and stationary phases (Figure 8C). The deletion of either the *mchQ* or *mchS* receptor genes does not influence the expression level of the *mch* gene cluster in both exponential and stationary phases. Thus, when *mchQ* is deleted the widening of the chelation halo observed earlier in CAS medium is not associated to an overexpression of the *mch* gene cluster, corroborating that mupirochelin is accumulating in the medium.

At last, RT-qPCR experiments were conducted on the siderophore-negative strain and its mutants in either *mchQ* or *mchS* supplemented with 10586∆pvdL∆mchAB∆trbABC (siderophore-negative) or 10586∆pvdL∆trbABC (mupirochelin-positive) supernatants in exponential phase (Figure 8B). Addition of mupirochelin-positive supernatant in 10586∆pvdL∆mchAB∆trbABC culture results in increasing expression levels of both *mchQ* and *mchS*. The same observation is made for the *mchQ* expression level in the 10586∆pvdL∆mchAB∆trbABC∆mchS mutant. However, supplementation of mupirochelin in the culture medium of the 10586∆pvdL∆mchAB∆trbABC∆mchQ mutant does not affect *mchS* level of expression. These results support the previous observation and suggest that MchQ is indeed the membrane receptor involved in mupirochelin uptake. Moreover, mupirochelin internalization appears to stimulate the biosynthesis of its uptake genes.

### Mupirochelin gene cluster transcription is regulated by iron availability

**Figure 9:**
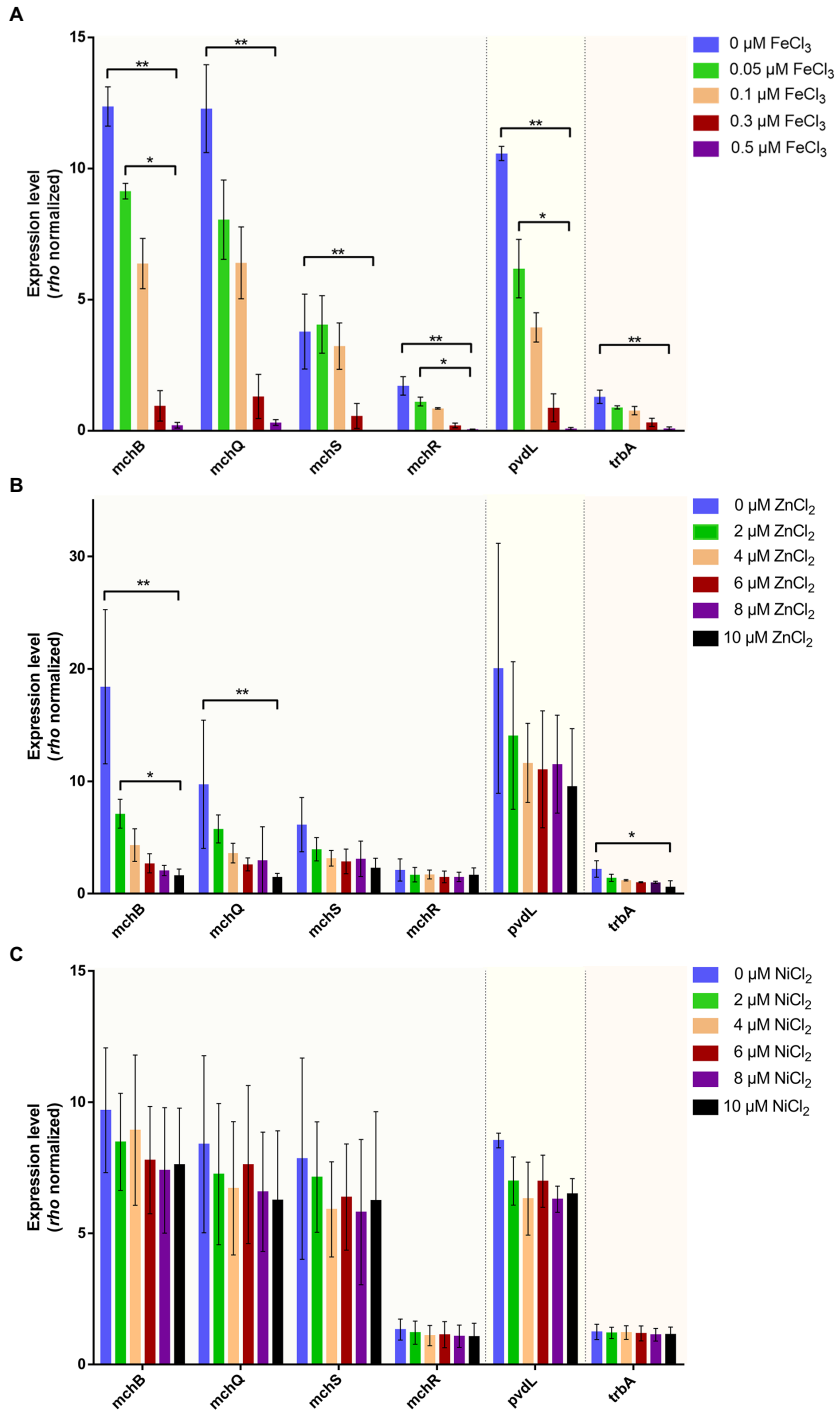
Effect of few metal concentrations on transcription for genes involved in pyoverdine (*pvdL*), mupirochelin (*mchB, mchQ, mchS, mchR*) and triabactin (*trbA*) pathways. **(A)** Increase in iron concentration in the culture medium is responsible for the repression of all the genes studied. At 0.5 μM FeCl_3_ the transcription of all genes is almost totally inhibited, even for *mchR* coding for the MchR transcriptional regulator of *mch*. **(B)** Increase in zinc concentration in the culture medium does not affect the transcription of *pvdL* indicating that pyoverdine may not have any specificity for Zn^2+^ ions. On the contrary, it appears that the expressions of *mchB* and *mchQ* follow the same trend and decrease with zinc concentration. Concentration in Zn^2+^ ion does not influence *mchR* gene transcription. *trbA* biosynthesis gene is also repressed by zinc concentration. **(C)** Increase in nickel concentration does not affect the expression level of all genes studied. All RT-qPCR analyses were conducted using *rho* as reference gene to study the level of expression of *pvdL* a biosynthetic gene of pyoverdine, *mchB* a biosynthetic gene of mupirochelin, *mchR* an AraC-like transcriptional regulator of *mch* cluster, *mchQ* and *mchS* coding TonB-dependent membrane receptors, and finally *trbA*, coding a siderophore synthase in the triabactin gene cluster (*n* ≥ 3). For a given gene, comparisons between groups were performed by Kruskal–Wallis test followed by Dunn’s multiple comparisons test *p < 0.05, **p < 0.01, ***p < 0.001.

The impact of a few metals (Fe^3+^, Zn^2+^, and Ni^2+^) on the transcription of operons of each siderophore was investigated. To this end, bacterial cultures were grown in CAA medium which were supplemented at the start of the exponential growth phase with different metal concentrations. After one hour of incubation, cells were harvested, and RNA was extracted for RT-qPCR analyses. As expected, the three siderophore gene clusters are repressed with increasing Fe^3+^ concentration in the medium, illustrating their role in iron homeostasis (Figure 9A). A concentration of 0.5 μM FeCl_3_ was sufficient to completely repress the transcription of pyoverdine (*pvdL*), triabactin (*trbA*) and mupirochelin (*mchB*) biosynthesis genes. This specific iron repression may be linked to Fur regulation (Troxell and Hassan, 2013) as putative Fur boxes were found in front of each studied operon (Supplementary Table S8).

Remarkably, in the presence of 10 μM Zn^2+^ transcription of *mchB, mchQ* and *mchS* is repressed, but to a lower extent than with 0.5 μM Fe^3+^, and the expression of *mchR* and *mchS* are not significantly affected by Zn^2+^(Figure 9B). Ni^2+^ concentration does not influence the transcription of all the studied genes (Figure 9C). Therefore, we hypothesized that the repression induced by iron and zinc ions suggests the ability of mupirochelin to chelate these ions.

**Figure 10:**
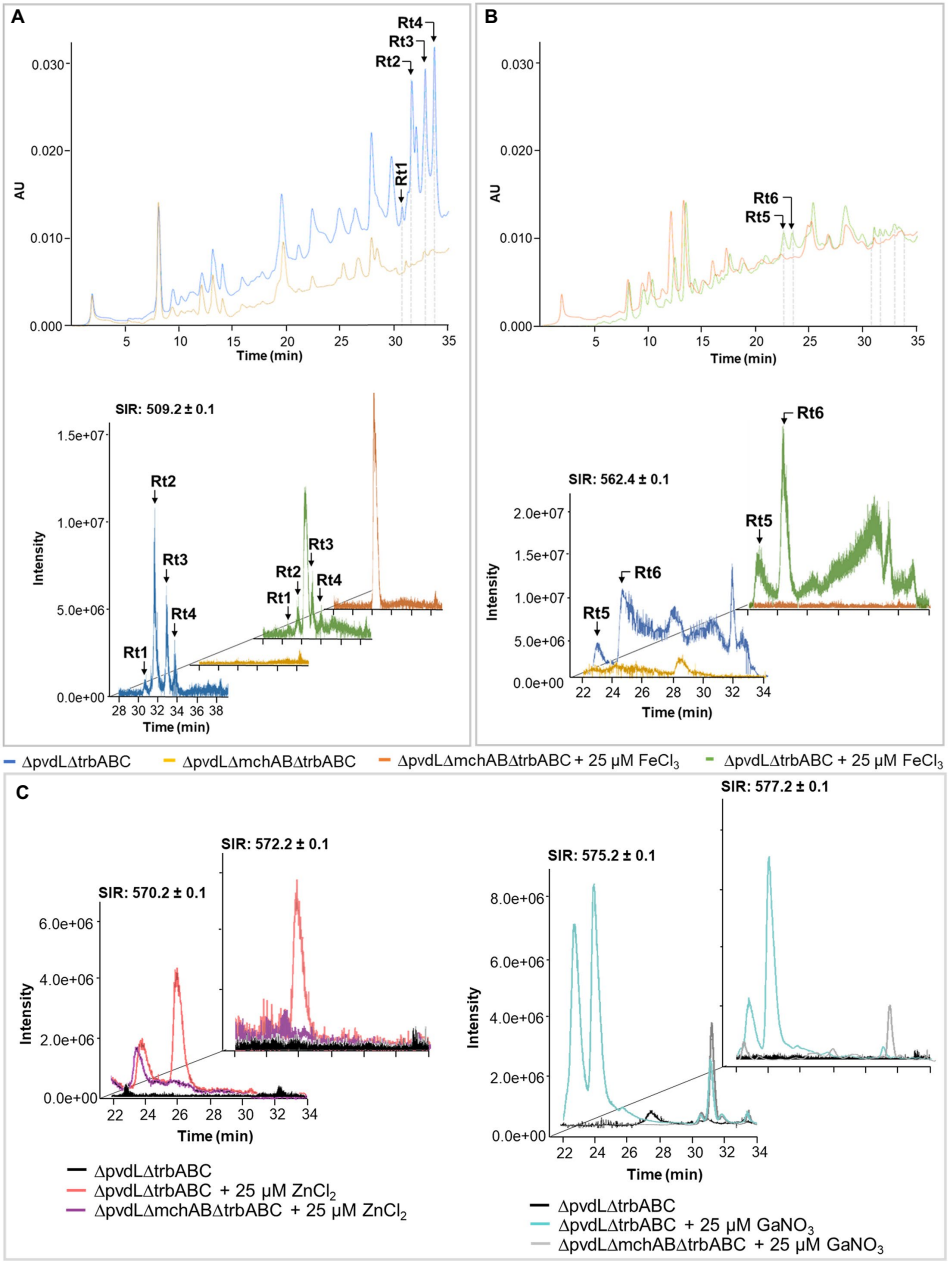
Evaluation of mupirochelin complexation with few metal ions by LC–MS analysis. **(A)** *Apo* mupirochelin (509.13 *m/z*) is characterized by a four-peaks signal at Rt1 = 30.673 min, Rt2 = 31.576 min, Rt3 = 32.836 min and Rt4 = 33.673 min. Addition of 25 μM FeCl_3_ is responsible for the loss of *apo* mupirochelin signal at 254 nm and its attenuation in MS detection. **(B)** Mupirochelin forms a complex with Fe^3+^ ions. Addition of FeCl_3_ to the sample is responsible for the appearance of two peaks at Rt5 = 22.567 min and Rt6 = 23.380 min. In mass detection, these two peaks are linked to the presence of 562.44 *m/z* analytes corresponding to the ferri-mupirochelin complex [M + Fe – 2H]^+^. **(C)** Mupirochelin chelates Zn^2+^ and Ga^3+^ ions. Addition of ZnCl_2_ to the sample (left) is responsible for the emergence of a peak mainly consisting of analytes 570.24 and 572.24 *m/z* suggested to be the zinc-mupirochelin complex. In the same way, addition of GaNO_3_ to the sample (right) implies the presence of two new peaks in the chromatogram with 575.21 and 575.24 *m/z* analytes corresponding to the gallium-mupirochelin complex.

To demonstrate the aptitude of mupirochelin to chelate these metals, FeCl_3_, GaNO_3_, ZnCl_2_, or NiCl_2_ salts, each at 25 μM, was added to the semi-purified supernatants of both the mupirochelin producing mutant 10586ΔpvdLΔtrbABC and the siderophore-negative mutant 10586ΔpvdLΔmchABΔtrbABC before analysis by LC–MS (Figure 10). *Apo* mupirochelin is eluted as four peaks at Rt1 = 30.67 min, Rt2 = 31.57 min, Rt3 = 32.83 min and Rt4 = 33.67 min (Figure 10A). After addition of 25 μM FeCl_3_ to the mupirochelin-positive semi-purified supernatant a notable reduction of mupirochelin signal is observed suggesting the complexation of the siderophore with Fe^3+^ ions (Figure 10B). Emergence of two peaks at Rt5 = 22.58 min and Rt6 = 23.38 min corresponding to analytes with 562.44 *m/z* in the chromatogram of 10586ΔpvdLΔtrbABC compared with 10586ΔpvdLΔmchABΔtrbABC could correspond to the formation of the ferri-mupirochelin complex ([M + Fe – 2H]^+^) (Figure 10B). Addition of 25 μM ZnCl_2_ to the supernatant results in the apparition of analytes with 570.24, 572.24, 573.31, and 574.39 *m/z* at 25.76 min and the disappearance of the *apo* mupirochelin signal suggesting the complexation of mupirochelin with zinc ions (^64^Zn, ^66^Zn, ^67^Zn, ^68^Zn) (Figure 10C). Similarly, the absence of mupirochelin signal and the appearance of two peaks at 22.60 and 24.18 min of analytes with 575.21 and 577.24 *m/z* when gallium is added to the sample could indicate the formation of mupirochelin complexes with gallium (^69^Ga and ^71^Ga) (Figure 10C). Finally, addition of NiCl_2_ does not affect the presence of *apo* mupirochelin in the sample indicating that the siderophore is not able to bind this metal.

### Expression of mupirochelin biosynthesis and uptake genes is affected by pyoverdine

The influence of pyoverdine and triabactin production on the regulation of mupirochelin biosynthesis was evaluated in NCIMB 10586 and its mutants. RT-qPCR assays were conducted on RNA of cells harvested during log-phase and stationary phase in CAA medium.

**Figure 11:**
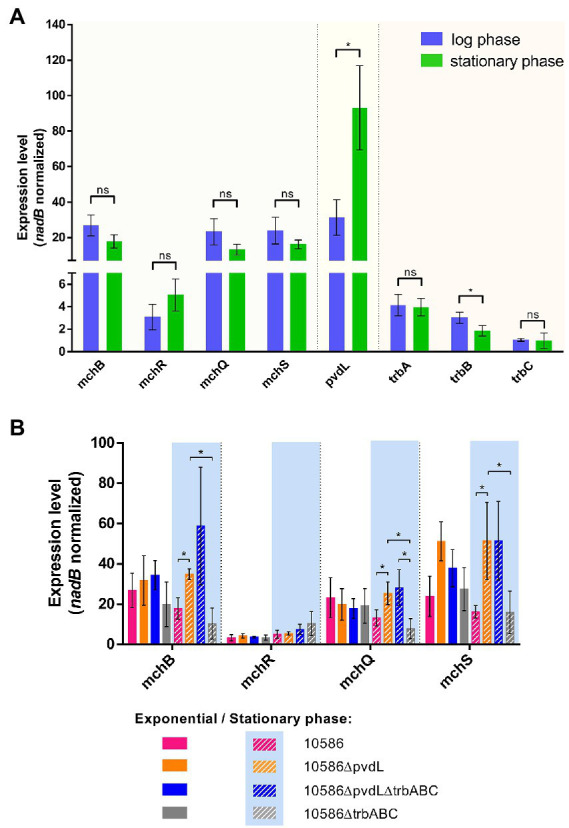
Influence of the growth phase on the expression of genes of the *mch* gene cluster. **(A)** Comparison of the expression levels of genes involved in pyoverdine, mupirochelin and triabactin pathways in the strain NCIMB 10586 during exponential and stationary growth phases. *pvdL* biosynthesis gene expression is about three times higher in stationary phase. The expression of mupirochelin and triabactin clusters is not influenced by bacterial growth (*n* = 4). Results of Mann–Whitney U-test for between-group pairwise comparison ns *p* > 0.05, *p < 0.05, **p < 0.01, ***p < 0.001. **(B)** Influence of pyoverdine and triabactin production on the *mch* gene cluster expression. In exponential phase, the levels of expression of all the studied genes appear constant between the different mutants. In stationary phase, *mchB, mchQ* and *mchS* levels of transcription are greater when *pvdL* is deleted (*n* = 3). Results of unpaired Student’s *t*-test with Welsh’s correction *p < 0.05, **p < 0.01, ***p < 0.001.

In the wild type, transcription of *pvdL* is about three times higher in stationary phase (Figure 11A). On the contrary, the expression of genes from the *mch* and *trb* clusters are not influenced by the bacterial growth stage. Thus, in the wild type triabactin and mupirochelin biosynthesis seem constant over time whereas pyoverdine production is clearly promoted in the early stationary phase.

In contrast, when pyoverdine biosynthesis is removed in strains 10586∆pvdL and 10586∆pvdL∆trbABC the expression of *mchB, mchQ*, and *mchS* tends to increase in stationary phase (Figure 11B). Thus, mupirochelin pathway regulation appears to be related to pyoverdine biosynthesis. On the contrary, transcription of the *trb* cluster is not affected by pyoverdine production (Supplementary Figure S6). Finally, absence of triabactin biosynthesis does not influence *mch* transcription.

## Discussion

Growth-inhibiting bacteria isolated from soil are a promising source of new natural compounds with interesting properties. The soil isolate *Pseudomonas* sp. NCIMB 10586 is well-known to produce the broad-range antibiotic mupirocin, a commercialized antibiotic active against Gram-positive and Gram-negative bacteria (Sutherland et al., 1985). In the current work, it has been shown that NCIMB 10586 is also active against an oomycete phytopathogen *Globisporangium* common in soil (Uzuhashi et al., 2010) through the action of the siderophore mupirochelin. In addition to mupirochelin NCIMB 10586 produces two more siderophores, the common pyoverdine PYO_13525_ (Matthijs et al., 2014) and triabactin identified in this study. Compared to both pyoverdine and triabactin, the production of mupirochelin does not confer a better fitness to the organism in *in vitro* iron-limited conditions, which raises the question of its primary biological function (Figure 6B).

Overall, mupirochelin (**1**) (Figure 3) appears to be homologous to the siderophores equibactin (**3**) and coelibactin (**4**) (Supplementary Figure S2). Mupirochelin is predicted to be an aryloxazoline-based siderophore with a linear structure consisting of five heterocycles, comprising two thiazolidine and one thiazoline rings. MS/MS analysis suggests the existence of at least five mupirochelin isomers in the semi-purified supernatant of *Pseudomonas* sp. NCIMB 10586 (Supplementary Figure S2). The aryloxazoline moiety is found in several siderophores and plays a crucial role in iron coordination and transport machinery (Kamińska et al., 2022). Besides, thiazoline and thiazolidine subunits arranged in tandem are encountered in diverse siderophore structures such as in compounds **2–8** (Supplementary Figure S3). These five-member rings can participate in the chelation of metal ions through their nitrogen atom. Based on its structure, the potential iron chelating motif of mupirochelin may involve the phenolic hydroxyl group, the nitrogen atoms of the oxazoline, thiazoline and thiazolidine rings, and the terminal carboxyl group. Thus, most of its chelating atoms are weak electron donors which could explain the low apparent affinity of mupirochelin toward iron (Figures 5C, 6).

Although mupirochelin does not seem to have a strong affinity toward iron, its ability to chelate Fe^3+^ ions (Figure 10) and the fine regulation by iron bioavailability of the genes involved in its pathway (Figure 9) suggest its importance in iron homeostasis. Moreover, in the wild type, in *in vitro* iron-limited conditions, mupirochelin and triabactin pathways transcription is constant over time, whereas pyoverdine biosynthesis gene transcription is clearly promoted to a later growth stage. Yet, when pyoverdine production is lost, the genes of mupirochelin pathway are overexpressed in the stationary phase, presumably to compensate for the loss of its most potent siderophore (see Figure 11).

Besides equibactin (**3**) and coelibactin (**4**), mupirochelin structure is homologous to the antifungal antibiotic transvalencin A (**9**) and the bioactive molecules uniformides A and B (**10–11**) (Supplementary Figure S3). Compounds **9** and **10** contain a zinc atom and corroborate the predicted iron chelating motif of mupirochelin. Here, LC–MS analysis suggests that mupirochelin is able to chelate Zn^2+^ ions (Figure 10). Moreover, excess of Zn^2+^ ions has been shown to repress the transcription of mupirochelin biosynthesis and transport genes (to a much lower extent than Fe^3+^), without affecting the expression level of *mchR* (Figure 9). A similar observation has already been made regarding the transcriptional regulation of the pyochelin gene cluster in *Pseudomonas aeruginosa* PAO1 (Carballido Lopez et al., 2019). Co^2+^ and Ni^2+^ ions were found to repress all the gene cluster, except for *pchR* gene coding for the AraC-like transcriptional regulator PchR. In the same study, PchR was demonstrated to activate the transcription of the gene cluster when interacting with the ferri-pyochelin complex. Subsequently, the repression observed was associated to PchR inability to activate the transcription when Co^2+^-pyochelin or Ni^2+^-pyochelin competed with Fe^3+^-pyochelin for PchR. We can therefore hypothesize that MchR, which activate the transcription of the *mch* gene cluster, plays a similar role as PchR (Figures 7–8). Therefore, the mild repression observed in presence of Zn^2+^ ions could be related to the competition between Zn^2+^-mupirochelin and Fe^3+^-mupirochelin for MchR. However, the difference between Zn^2+^ and Fe^3+^ concentration needed to observe a similar repression underlines the specificity for iron of the *mch* cluster regulation (see Figure 9).

If sideromycins are a well-known class of antibiotics, combining a bactericidal molecule and a siderophore part, little is known about true siderophores with antimicrobial activity independent from iron competition. Nocardamin and friadamine A and B are siderophores recognized to have some moderate antibiotic activities, and thioquinolobactin is known to inhibit *Globisporangium* growth (Stoll et al., 1951; Matthijs et al., 2007; Takehana et al., 2017). Here, the anti-*Globisporangium* activity of mupirochelin is not directly related to iron competition since both efficient iron chelators pyoverdine and triabactin are not able, alone or combined, to impede the proliferation of the oomycete. However, the single production of pyoverdine is responsible for the reduction of the hyphal density around the colony compared to the siderophore-negative mutant. Furthermore, additional production of pyoverdine by the mupirochelin producer steps up the growth inhibitory activity of the strain against the phytopathogen (Supplementary Figure S1A). These results suggest that the iron competition generated by pyoverdine associated with the original antagonism activity of mupirochelin have a synergetic activity against the oomycete. Besides, we show that the antagonism activity of mupirochelin is lost in its complexed state with Fe^3+^ or Ga^3+^ ions. Here, gallium(III) is used as a mimic of iron(III) based on their similar atomic radius, electronic configuration, charge state and coordination chemistry. Iron release from siderophores is mediated through the reduction of Fe^3+^ to Fe^2+^, but the trivalent gallium cannot be reduced to a divalent state (Bernstein, 1998) and, therefore, is able to lock the siderophore in its complexed form. Thus, mupirochelin antagonism activity against the oomycete stem from its conformational state. Since, oxazoline and thiazoline rings have been shown to be important for biological activity, these five-member heterocycles could be responsible for the observed *in vitro* antagonism (Kakkar and Narasimhan, 2019; Arshad et al., 2022).

The presence of two different TonB-dependent membrane receptors in the gene cluster of a siderophore is not new but rather unusual (Cornelis and Bodilis, 2009; Ye et al., 2014). For the mupirochelin gene cluster, the receptor coding genes *mchQ* and *mchS* are almost always found together. Of the 114 strains that have a *mch* gene cluster in their genome, only three closely related strains miss the *mchS* gene. Further study of these strains is needed as it is currently not known whether they produce mupirochelin. In addition, the strains of the *P. gessardi* subgroup that miss the biosynthetic operon and that are expected to take up mupirochelin, always have both TonB-dependent receptors in their gene cluster. These observations suggest that both receptors are needed. Results presented in this work indicate that MchQ is the receptor for the uptake of mupirochelin, whereas the function of the second receptor MchS is not clear. The *mchS* gene is not silent under the studied conditions: its expression is stimulated by mupirochelin uptake and is iron-repressed, therefore suggesting its implication in iron metabolism. However, its sequence divergence from MchQ (amino acid identity of 55%) tends to indicate that it may be involved in the transports of a distinct ligand.

The plant growth promoting rhizobacteria (PGPR) *P. simiae* WCS417 (Berendsen et al., 2015) and many strains of the plant-associated *P. corrugata* subgroup are predicted to produce mupirochelin, based on the presence of the mch gene cluster in their genome (Supplementary Figure S4). Whether the secretion of this siderophore by plant-associated strains could be linked to its anti-oomycete activity and provide plant protection awaits further investigation.

Finally, the siderophore triabactin seems to have a good affinity for iron as it can improve strain fitness in *in vitro* iron-stressed conditions. The *trb* gene cluster is rather simple, comprising only one operon formed by *trbA* involved in the biosynthesis of the siderophore, *trbB* involved in its secretion, and *trbC* involved in its uptake by the strain. Under the studied conditions, the iron concentration in the environment was the only parameter demonstrating a specific regulatory effect on the transcription of this gene cluster (Figure 9). The presence of a putative Fur-box upstream the *trb* gene cluster supports this observation (Supplementary Table S8). Further work on this siderophore will consist in its purification and the resolution of its structure.

Production and utilization of multiple siderophores by bacteria has already been observed in the literature giving rise to various hypotheses regarding the usefulness of redundant siderophore-mediated iron uptake systems. One common hypothesis regarding this overlap is about differential specificity for iron under different environmental conditions enhancing the degree of fitness of the organism (Johnstone and Nolan, 2015; Katumba et al., 2022). Here, under the studied conditions, we show that the chronology of production and chelating activity are siderophore-dependent. Triabactin is presumed to be a rather simple, yet potent, siderophore that originates from the modification of a primary metabolite such as citrate. Its biosynthesis is rather constant over time. Pyoverdine, a strong and complex siderophore, is mostly produced in the early stationary phase. Finally, biosynthesis of the weak siderophore mupirochelin is constant over time in the wild type but is promoted in stationary phase when pyoverdine is absent. Based on the results obtained in this study, it is noteworthy to point out that mupirochelin production can be improved by inactivation of pyoverdine and MchQ biosynthesis. The production of these three compounds is suggested to be Fur-mediated which corroborates their implication in iron metabolism. This production of multiple siderophores is a major asset in iron-limited conditions and greatly contributes to the strain fitness. Moreover, numerous other secondary metabolites are predicted to be produced by the strain. However, as for mupirochelin and triabactin, some of these compounds have not been identified yet. Their nature and physiological role remain to be investigated to unravel their own contribution to the strain fitness (Supplementary Table S9).

In conclusion, we identified two novel siderophores produced by pseudomonads such as *Pseudomonas* sp. NCIMB 10586, namely the NRPS-dependent siderophore mupirochelin and the NIS triabactin. The aryloxazoline siderophore mupirochelin shares structural similarities with other siderophores and with some bioactive natural compounds. Apart from its chelating properties, mupirochelin has also been found to inhibit *Globisporangium* growth in a way that does not seem to be related to competition for iron. However, this growth inhibiting activity is lost when the siderophore is chelating a metal ion such as Fe^3+^ or Ga^3+^. To assess the biocontrol ability of the pseudomonads producing mupirochelin, further work needs to be done *in vitro* and *in planta.* In particular, the mechanism of growth inhibition remains uncharacterized yet and must be investigated in order to have a better understanding of the mechanism of resistance that could arise from mupirochelin use.

## Data availability statement

The raw data supporting the conclusions of this article will be made available by the authors, without undue reservation.

## Author contributions

CG conducted most of the laboratory experiments, data analysis, and the manuscript write-up. NB performed the RT-qPCR experiments. PA performed the LC-MS/MS analysis and predicted the structure of mupirochelin. RW assisted in data analysis. SM carried out the phylogenetic analysis and designed and supervised the entire study. All authors proofread and reviewed the manuscript.

## Funding

CG is a FRIA grantee and RW a Research Associate of the Fonds de la Recherche Scientifique – FNRS, Belgium. The Analytical Platform is supported by the FNRS and Université libre de Bruxelles.

## Conflict of interest

The authors declare that the research was conducted in the absence of any commercial or financial relationships that could be construed as a potential conflict of interest.

## Publisher’s note

All claims expressed in this article are solely those of the authors and do not necessarily represent those of their affiliated organizations, or those of the publisher, the editors and the reviewers. Any product that may be evaluated in this article, or claim that may be made by its manufacturer, is not guaranteed or endorsed by the publisher.

## Footnotes

^1^ https://inp.univ-amu.fr/en/amplifx-manage-test-and-design-your-primers-for-pcr
