# Supplementary material for: Two new siderophores produced by Pseudomonas sp. NCIMB 10586: The anti-oomycete non-ribosomal peptide synthetase-dependent mupirochelin and the NRPS-independent triabactin
Source: Front Microbiol. 2023 Mar 24;14:1143861. doi: 10.3389/fmicb.2023.1143861 (PMC10080011; doi:10.3389/fmicb.2023.1143861)
Supplement: Supplementary file 1 [file Data_Sheet_1.pdf]

Supplementary Material

Supplementary Table 1. Plasmids used in this study.

**Plasmids**	**Characteristics**	**Reference**
pTnModO-Tc	Plasposon, Tn5 transposase, R6K, Tc^R	Dennis & Zylstra 1998
pUK21	Cloning vector, 3090 bp, ColE1 replicon, Km^R, *lacZ*	Vieira & Messing 1991
pUK-pvdL	pUK21 containing the 5′ and 3′ end of *pvdL*; Km^R	This study
pUK-mchAB	pUK21 containing the 5′ of *mchA* and 3′ end of *mchB*, Km^R	This study
pUK-trbABC	pUK21 containing the 5′ upstream region of *trbA* and 3′ downstream region of *trbC*, Km^R	This study
pUK-mchQ	pUK21 containing the 5′ and 3′ flanking regions of *mchQ*, Km^R	This study
pUK-mchS	pUK21 containing the 5′ and 3′ flanking regions of *mchS*, Km^R	This study
pUK-mchR	pUK21 containing the 5′ and 3′ flanking regions of *mchR*, Km^R	This study
pME3087	Suicide vector, 6,822 bp, ColE1 replicon, RK2-Mob, Tc^R	Voisard et al. 1994
pME-pvdL	pME3087 containing the 5′ and 3′ end of *pvdL*; Tc^R	This study
pME-mchAB	pME3087 containing the 5′ of *mchA* and 3′ end of *mchB*; Tc^R	This study
pME-trbABC	pME3087 containing the 5′ upstream end of *trbA* and 3′ downstream end of *trbC*; Tc^R	This study
pME-mchQ	pME3087 containing the 5′ and 3′ flanking regions of *mchQ*; Tc^R	This study
pME-mchS	pME3087 containing the 5′ and 3′ flanking regions of *mchS*; Tc^R	This study
pME-mchR	pME3087 containing the 5′ and 3′ flanking regions of *mchR*; Tc^R	This study

References:

Dennis, J. J., and Zylstra, G. J. (1998). Plasposons: modular self-cloning minitransposon derivatives for rapid genetic analysis of gram-negative bacterial genomes. Appl. Environ. Microbiol. 64, 2710–2715. doi: 10.1128/aem.64.7.2710-2715.1998

Vieira, J., and Messing, J. (1991). New pUC-derived cloning vectors with different selectable markers and DNA replication origins. Gene 100, 189–194. doi: 10.1016/0378-1119(91)90365-I

Voisard, C., Bull, C., Keel, C., Laville, J., Maurhofer, M., Schnider, U., et al. (1994). "Biocontrol of root diseases by *Pseudomonas fluorescens* CHA0: current concepts and experimental approaches" in Molecular Ecology of Rhizosphere Microorganisms. eds. F. O'Gara, D. Dowling and B. Boesten (VCH: Weinheim), 67–89

Supplementary Table 2. Predicted functions of the proteins encoded by the different *mch* genes based on the presence of motifs similarities (InterPro).

ORF (length)	Domain localization	InterPro entry	Domain prediction	Predicted protein
MchA (2135 aa)	9-63	IPR020806	Polyketide synthase, phosphopantetheine-binding domain	Non-ribosomal peptide synthetase
	129-517	IPR001242	Condensation domain	
	574-950	IPR010071	Amino acid adenylation domain	
	1027-1102	IPR009081	Phosphopantetheine binding ACP domain	
	1155-1457	IPR001242	Condensation domain	
	1590-1978	IPR010071	Amino acid adenylation domain	
	1962-2028	IRP025110	AMP-binding enzyme, C-terminal domain	
	2056-2125	IPR020806	Polyketide synthase, phosphopantetheine-binding domain	
MchB (1817 aa)	5-53	IPR041464	TubC, N-terminal docking domain	Non-ribosomal peptide synthetase
	97-493	IPR001242	Condensation domain	
	534-925	IPR000873	AMP-dependent synthetase/ligase	
	1165-1265	IPR013217	Methyltransferase type 12	
	1404-1485	IPR009081	Phosphopantetheine binding ACP domain	
	1519-1622	IPR001031	Thioesterase	
MchC (579 aa)	18-278	IPR011527	ABC transporter type 1, transmembrane domain	ABC transporter ATP-binding protein
	333-566	IPR003439	ABC transporter-like, ATP-binding domain	
MchD (581 aa)	21-299	IPR011527	ABC transporter type 1, transmembrane domain	ABC transporter ATP-binding protein
	313-565	IPR003439	ABC transporter-like, ATP-binding domain	

MchE (367 aa)	3-107	IPR005097	Saccharopine dehydrogenase, NADP binding domain	Saccharopine dehydrogenase
MchF (342 aa)	166-266	IPR013217	Methyltransferase type 12	Methyltransferase
MchG (558 aa)	347-447	IPR013217	Methyltransferase type 12	Methyltransferase
MchH (392 aa)	172-363	IPR001128	Cytochrome P450	Cytochrome P450
MchI (379 aa)	7-123	IPR000683	Gfo/Idh/MocA-like oxidoreductase, N-terminal	Thiazolinyl imide reductase
MchJ (239 aa)	12-186	IPR025870	Glyoxalase-like domain	Hypothetical protein
MchK (532 aa)	31-435	IPR000873	AMP-dependent synthetase/ligase	2,3-dihydroxybenzoate-AMP ligase
	444-519	IPR025110	AMP-binding enzyme, C-terminal domain	
MchL (248 aa)	17-236	IPR001031	Thioesterase	Thioesterase
MchM (102 aa)	4-94	IPR002701	Chorismate mutase II, prokaryotic-type	Isochorismate-pyruvate lyase
MchN (475 aa)	187-444	IPR015890	Chorismate-utilising enzyme, C-terminal	Isochorismate synthase
MchO (294 aa)	7-237	IPR003439	ABC transporter-like, ATP-binding domain	ABC transporter ATP-binding protein
MchP (1020 aa)	-	-	-	Hypothetical protein
MchQ (715 aa)	67-172	IPR012910	TonB-dependent receptor, plug domain	TonB-dependent receptor
	264-714	IPR000531	TonB-dependent receptor-like, beta-barrel	

MchR (316 aa)	209-307	IPR018060	DNA binding HTH domain, AraC-type	AraC family transcriptional regulator
MchS (707 aa)	58-163	IPR012910	TonB-dependent receptor, plug domain	TonB-dependent receptor
	264-704	IPR000531	TonB-dependent receptor-like, beta-barrel	

Supplementary Table 3. The primers, and their sequence, used for the amplification of fragment A and B for the construction of plasmid pUK-pvdL, pUK-mchAB, pUK-trbABC, pUK-mchQ, pUK-mchS, pUK-mchR are given. The restriction sites are underlined.

Primer name	Primer sequence (5'–3')
**Amplification fragment A (AF-AR) and B (BF- R):**	
ΔpvdL-AF	GTGAAGCTTATGATGGACGCCTTCGAACT
ΔpvdL-AR	GTGTCTAGAGCGCTTGCTCGTCCATGT
ΔpvdL-BF	GTGTCTAGACGAACACCATTGCCCATGA
ΔpvdL-BR	GTGGAATTCCCTCCAACTCCGCCATCA
ΔmchAB-AF	GTGAAGCTTCCCAGCACCTCAACCCTAAC
ΔmchAB-AR	GTGTCTAGAGTTGAACAGCGGCAGGTTGA
ΔmchAB-BF	GTGTCTAGAGGTGCCGACGACAGCTTCT
ΔmchAB-BR	GTGGGATCCGTATCGAGTGCGGTGCTCATGA
ΔtrbABC-AF	GTGGGATCCTTGCCCTGCGCCAGGTTGA
ΔtrbABC-AR	GTGTCTAGACCTGCTATTTCCGTGCATCT
ΔtrbABC-BF	GTGTCTAGAAGTTACGCAGTGGATTGGTAAC
ΔtrbABC-BR	GTGGAATTCGCCCGTCACCTCAAGGTTGA
ΔmchQ-AF	GTGAAGCTTCCTCAACGGCGCACTGTTCT
ΔmchQ-AR	GTGTCTAGAGTGGGGTATAGCTATCAGTTCT
ΔmchQ-BF	GTGTCTAGAAGATGACATGGCAAAGCTCCAA
ΔmchQ-BR	GTGGGATCCCGGTTGTCAGGTGGTGTTGA
ΔmchS-AF	GTGAAGCTTGGCTGCGCGCCTGGATCA
ΔmchS-AR	GTGTCTAGAGCAGACATGATGGGACCTTGA
ΔmchS-BF	GTGTCTAGAGGGCTATACCTATCAATTCTGAA
ΔmchS-BR	GTGGGATCCCTTCAGTGTGGCCTGGAAGA
ΔmchR-AF	GTGGGATCCGGTTAGTCGTGCCACGAAGT
ΔmchR-AR	GTGGTCGACGGGCGGTAGGGTTTTCATTG
ΔmchR-BF	GTGGTCGACGCATGCTTGCCAGCTTGATCT
ΔmchR-BR	GTGTCTAGACAGCCCTGCGCATCCCATTG
**Deletion confirmation:**	

ΔpvdL-delF	GAGGAAATCAGCCGTGATCT
ΔpvdL-delR	CCCTCGTACCATACGATCA
ΔmchAB-delF	GGCTCTACCCGATTCAAGT
ΔmchAB-delR	GTGTGGCTGTGGGCATTG
ΔtrbABC-delF	TCAACGTCACCCTCGAAAGC
ΔtrbABC-delR	CACCGCCTTACCTTCGTTGT
ΔmchQ-delF	GCACTCACCGACCACTATG
ΔmchQ-delR	CGGCCTCGATGCCGAGTT
ΔmchS-delF	GACGGATTGATCCCGAACT
ΔmchS-delR	CTGCTGGGGTATGACTTCA
ΔmchQΔmchS -delF	GCACTCACCGACCACTATG
ΔmchQΔmchS -delR	CCTGTGCAATGAAGTTGAA
ΔmchR-delF	GATCGTCAGCGGCTGATGA
ΔmchR-delR	GCGCCCTTGGTAGTGTACA

Supplementary Table 4. The primers and their sequence used for RT-qPCR analysis.

Primer name	Primer sequence (5'–3')	R^2	Efficiency
algD-F	TGGTCGGCGTAGACATCTC	1.00	2.05
algD-R	TACCCTGGCTCAACAGCTC		
fabD-F	GCCTCGATTACCACCGCAT	1.00	1.96
fabD-R	TGGTGAAGCGGTCAAGCTG		
gyrA-F	GCTGGGTAACGACTGGAAC	1.00	1.97
gyrA-R	CCATCCGAACGATGGTGTC		
mchB-F	CGAGTTTGTCCACGGCATT	1.00	1.97
mchB-R	CAGCGTATTGCGTGCTGAA		
mchQ-F	CGCCTTCGCTTTCATGCTT	1.00	1.93
mchQ-R	ACTACGCCACCTCGATCAA		
mchR-F	ATTGCCGGGTCACGCGTAT	1.00	1.98
mchR-R	TGAATGCACTCGCTGTCGT		
mchS-F	ACGACAGCTGGATCGACAA	1.00	1.97
mchS-R	AACAGCACGTGGGTGTCAT		
nadB-F	TGATCGACCAAGGTGTGCC	1.00	1.95
nadB-R	TTCGCGTGTCAGGTGGAA		
oprL-F	GGTAAGTTTGCTGCTCTGG	1.00	2.03
oprL-R	GCGTTTGGATCAACAGCT		
pks-F	TCAGGCCTTCAAGTAGCTCG	1.00	1.98
pks-R	CAAATCGCGCTTCGACCTG		
pvdL-F	AAAGCACCGTGCAAGGCAT	1.00	1.99
pvdL-R	TCGCCAATCAGCGGCAAAT		
rho-F	GAAAATATGGCCCGTTCGCG	1.00	1.97
rho-R	AATCTCCAGCACGCCATCAC		
rpsL-F	CGAGAAATCCGACGTACCT	1.00	1.95
rpsL-R	CACGGCATACTTTACGCAG		
trbA-F	CGGAAATAGCAGGAAGCCG	1.00	2.01
trbA-R	CCAGCGCATTGACCAAGT		

trbB-F	ATCCGGTTTCGCATTGCT	1.00	1.98
trbB-R	TGAATCGCTGGGCAAGAAC		
trbC-F	AGCAGAACGGCAAATGGA	1.00	2.04
trbC-R	ACGCTGATAGCCGACAAA		

Supplementary Table 5. Ranking of six reference genes by RefFinder tool for *Pseudomonas* sp. NCIMB 10586 cultures grown in CAA medium and CAA medium supplemented with 0.5 µM $FeCl_3$, 10 µM $ZnCl_2$ or 10 µM $NiCl_2$. *algD*: GDP-mannose 6-dehydrogenase, *fabD*: malonyl CoA-acyl carrier protein transacylase, *gyrA*: DNA gyrase subunit A, *oprL*: peptidoglycan-associated protein, *rho*: transcription termination factor Rho, *rpsL*: 30S ribosomal protein S12.

Method	1	2	3	4	5	6
Delta CT	rho	algD	oprL	fabD	gyrA	rpsL
BestKeeper	algD	rho	oprL	fabD	rpsL	gyrA
NormFinder	rho	algD	oprL	fabD	gyrA	rpsL
Genorm	algD / rho		oprL	fabD	gyrA	rpsL
Recommended comprehensive ranking	rho	algD	oprL	fabD	gyrA	rpsL

Supplementary Table 6. Ranking of six reference genes by RefFinder tool for *Pseudomonas* sp. NCIMB 10586 cultures harvested in exponential and stationary phases. *algD*: GDP-mannose 6-dehydrogenase, *gyrA*: DNA gyrase subunit A, *nadB*: L-aspartate oxidase, *oprL*: peptidoglycan-associated protein, *pks*: polyketide synthase, *rpsL*: 30S ribosomal protein S12.

Method	1	2	3	4	5	6
Delta CT	nadB	gyrA	pks	algD	rpsL	oprL
BestKeeper	nadB	gyrA	algD	pks	rpsL	oprL
NormFinder	nadB	gyrA	pks	algD	rpsL	oprL
Genorm	gyrA / nadB		pks	algD	rpsL	oprL
Recommended comprehensive ranking	nadB	gyrA	pks	algD	rpsL	oprL

Supplementary Table 7. Strains from our in-house *Pseudomonas* collection analyzed by LC-MS for mupirochelin production.

**Strains**	**Relevant characteristics**	**Reference**
Pseudomonas azotoformans LMG 23662^{T}	Mupirochelin producer	BCCM/LMG
Pseudomonas canadensis LMG 28499^{T}	Mupirochelin producer	BCCM/LMG
Pseudomonas libanensis LMG 21606^{T}	Mupirochelin producer	BCCM/LMG
Pseudomonas simiae DSM 18861^{T}	Mupirochelin producer	DSMZ
Pseudomonas synxantha DSM 18928^{T}	Mupirochelin producer	DSMZ
Pseudomonas thivervalensis LMG 21626^{T}	Mupirochelin producer	BCCM/LMG
Pseudomonas sp. W2Jun17	Mupirochelin producer	Matthijs et al., 2013
Pseudomonas sp. W2Aug9	Mupirochelin producer	Matthijs et al., 2013

Supplementary Table 8. Putative Fur-boxes found in the promoter sequences of genes coding for the three siderophores under study. Asterisks indicate the consensus motif for one strain in comparison with the model Fur box from *P. aeruginosa*.

**Operon**																				**Id. with the closest confirmed Fur-box sequence**	**Source**
P. NCIMB 10586	*			*				*			*	*		*		*	*				
pvdL	G	A	C	A	A	T	C	A	T	T	A	T	C	A	T	T	A	A	G	73% (14/19)	Ochsner and Vasil 1996
trbABC	G	A	T	A	T	C	G	A	G	A	A	T	C	A	T	T	A	T	T	68% (13/19)	Wilderman et al. 2004
mchR	G	C	A	A	T	T	G	A	T	A	A	T	T	A	T	T	A	G	C	73% (14/19)	Ochsner and Vasil 1996
mchABCDEFGHIJKLMN	G	A	C	A	A	T	G	A	G	A	A	T	G	A	C	T	A	G	C	68% (13/19)	Elias et al. 2011
A. dieselolei DSM 16502				*	*	*	*	*		*	*		*	*	*	*	*	*	*		
mchABCDEFGHIJKLMN	A	C	A	A	A	T	G	A	G	A	A	C	C	A	T	T	A	T	C	79% (15/19)	Wilderman et al. 2004
L. anthtropi DSM 23190	*	*	*	*	*	*			*		*	*		*	*	*			*		
mchABCDEFGHIJKLMN	G	A	T	A	A	T	A	G	T	T	A	T	G	A	T	T	T	G	C	68% (13/19)	Ochsner and Vasil 1996
Model (*P. aeruginosa*)	G	A	T	A	A	T	G	A	T	A	A	T	C	A	T	T	A	T	C		Ochsner and Vasil 1996

References:

Elias S., Degtyar E., Banin E (2011). FvbA is required for vibriobactin utilization in *Pseudomonas aeruginosa*. Microbiology 157:2172-2180. Doi: 10.1099/mic.0.044768-0

Ochsner U.A., Vasil M.L. (1996). Gene repression by the ferric uptake regulator in *Pseudomonas aeruginosa*: Cycle selection of iron-regulated genes. Proc. Natl. Acad. Sci. 93:4409-4414. Doi: 10.1073/pnas.93.9.4409

Wilderman P.J., Sowa N.A., FitzGerald A.J., FitzGerald P.C., Gottesman S., Ochsner U.A., Vasil M.L. 2004. Identification of tandem duplicate regulatory small RNAs in *Pseudomonas aeruginosa* involved in iron homeostasis. Proc Natl Acad Sci. 101(26), 9792-9797. Doi: 10.1073/pnas.0403423101

Supplementary Table 9. Secondary metabolite gene clusters in *Pseudomonas* sp. NCIMB 10586 predicted with antiSMASH tool version 6.0. NIS: NRPS-independent siderophore, NRPS: Non-ribosomal peptide synthase, RiPP-like: Unspecified ribosomally synthesized and post-translationally modified peptide product, NANGGN: N-acetylglutaminylglutamine amide, PKS: Polyketide synthase

Cluster n°	Coordinates	Type	Most similar known cluster	Similarity	Predicted metabolite
1	111,591-174,501	NRP-metallophore, NRPS	pyochelin	28%	mupirochelin
2	251,130-327,878	NRP-metallophore, NRPS	Pf-5 pyoverdine	11%	pyoverdine
3	1,002,824-1,015,064	RiPP-like	lipopolysaccharide	5%	-
4	1,283,211-1,344,379	NRP	viscosin	43%	viscosin
5	1,521,611-1,549,511	NRP, betalactone	fengycin	13%	-
6	1,673,711-1,718,401	NRPS	MA026	14%	-
7	1,966,949-1,981,826	NRP, NAGGN	-	-	-
8	2,016,802-2,069,698	NRPS	Pf-5 pyoverdine	9%	pyoverdine
9	2,159,382-2,171,307	NRP, NIS	-	-	triabactin
10	3,344,358-3,366,505	NRP + PKS, redox-cofactor	lankacidin	13%	-
11	4,023,191-4,066,568	NRPS-like	ambactin	25%	-
12	4,375,844-4,419,419	Other, arylpolyene	arylpolyene	40%	arylpolyene
13	5,882,478-5,985,258	Iterative type I polyketide Trans-AT type I polyketide	pseudomonic acid A	100%	pseudomonic acid A

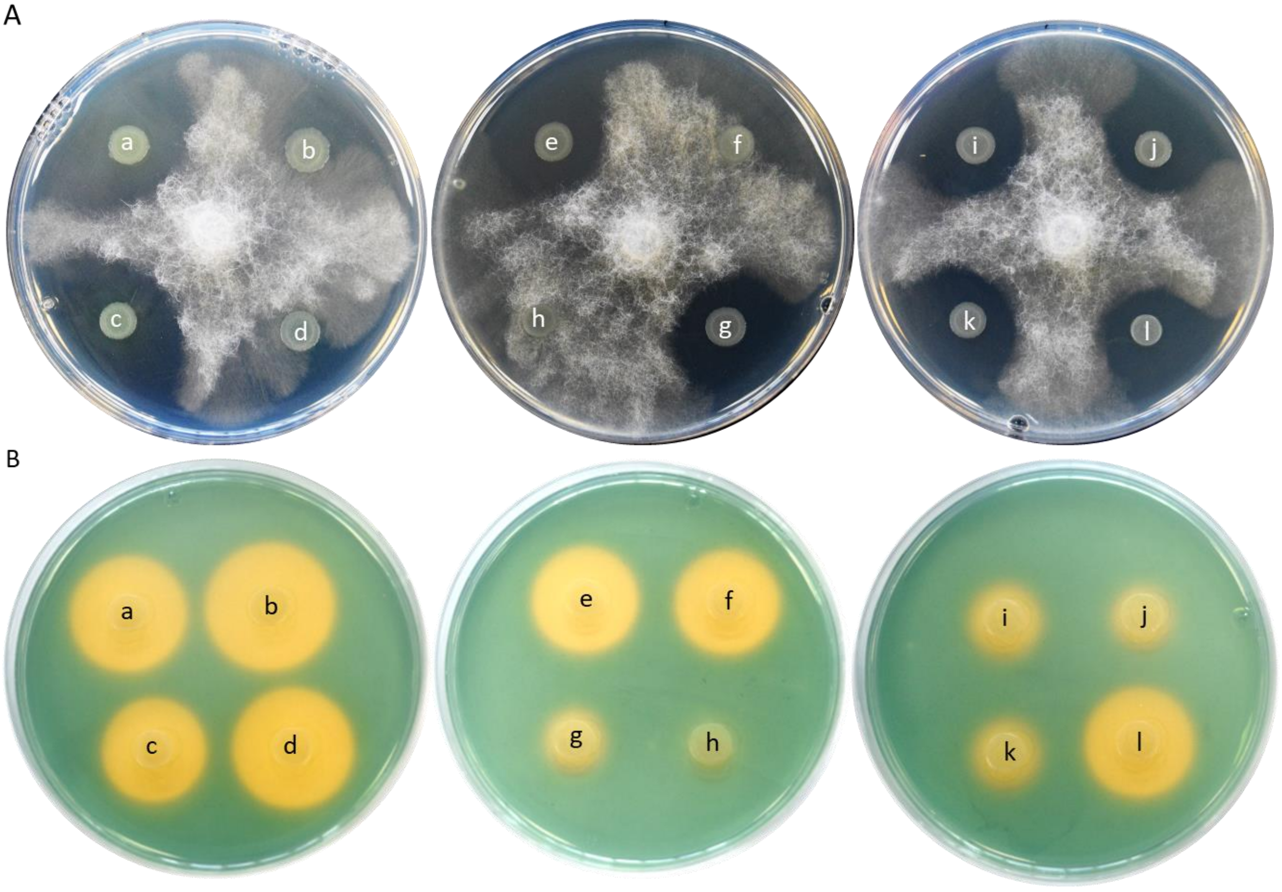

Supplementary Figure 1. Characterization of *Pseudomonas* sp. NCIMB 10586 and its mutants. **(A)** *In vitro* antagonism assay of each strain against *G. ultimum* MUCL 38045. Mupirochelin-producing strains inhibit the growth of the oomycete (a, c, e, g, i, j, k and l). In a mupirochelin-negative background, the production of pyoverdine slows down the hyphal progression on the medium (b, d). Moreover, the combined production of pyoverdine and mupirochelin tends to step up the growth inhibition zone (a and c). Furthermore, the production of triabactin in a pyoverdine/mupirochelin-negative background is also responsible for the slowdown of the hyphal progression (f). **(B)** Comparison of siderophore production on CAS medium. A change in color from blue-green to yellow-orange suggests the production of a chelator in the medium. Production of pyoverdine and/or triabactin leads to a large halo around the colony (a, b, c, d e, f and l). Production of only mupirochelin gives a small halo (g, i, j and k). Loss of the biosynthesis of the three siderophores results in complete loss of CAS activity (h).

a: wild type, b: 10586ΔmchAB, c: 10586ΔtrbABC, d: 10586ΔmchABΔtrbABC, e: 10586ΔpvdL, f: 10586ΔpvdLΔmchAB, g: 10586ΔpvdLΔtrbABC, h: 10586ΔpvdLΔmchABΔtrbABC, i: 10586ΔpvdLΔtrbABCΔmchQ, j: 10586ΔpvdLΔtrbABCΔmchS, k: 10586ΔpvdLΔtrbABCΔmchQΔmchS, l: 10586ΔpvdLΔmchR.

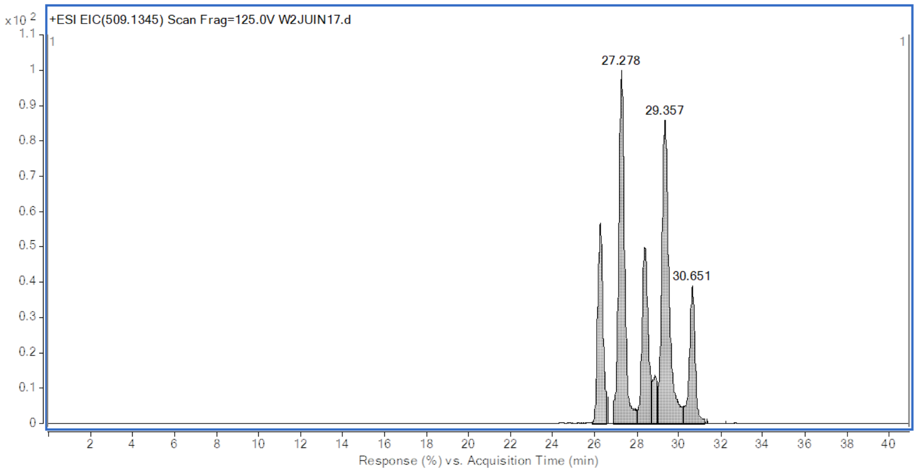

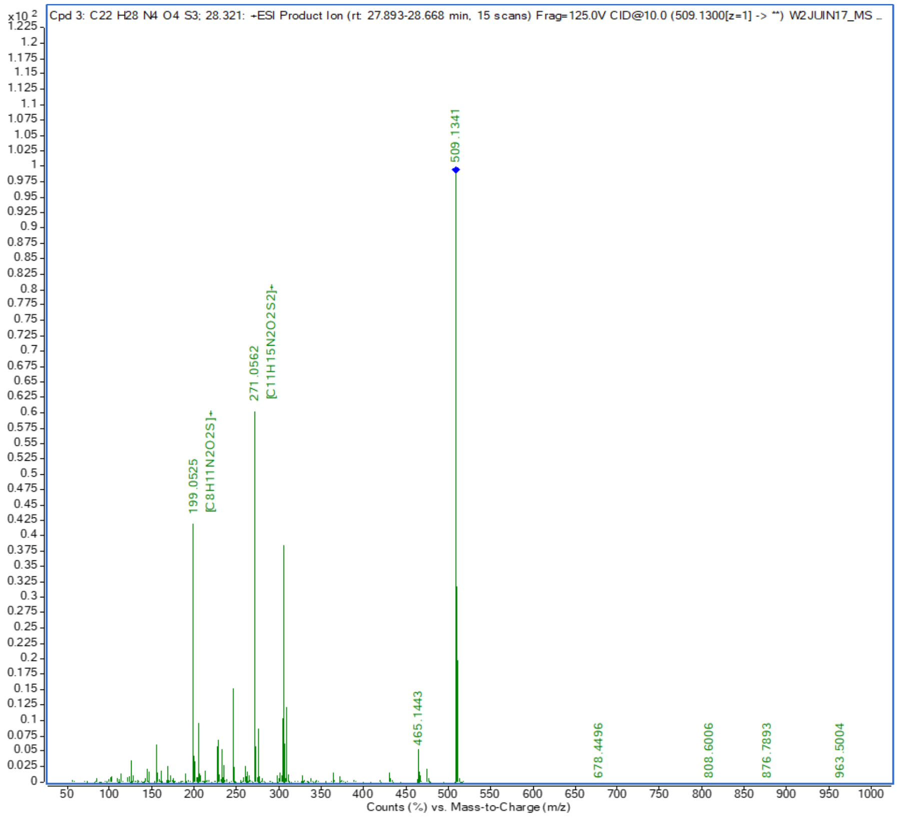

Supplementary Figure 2. Extracted chromatogram from LC-MS injection of the mupirochelin-containing supernatant. The chromatogram shows 5 different peaks at 509.1345 *m/z* absent from the supernatant of the mupirochelin-negative supernatant which are postulated as 5 isomers of the same structure.

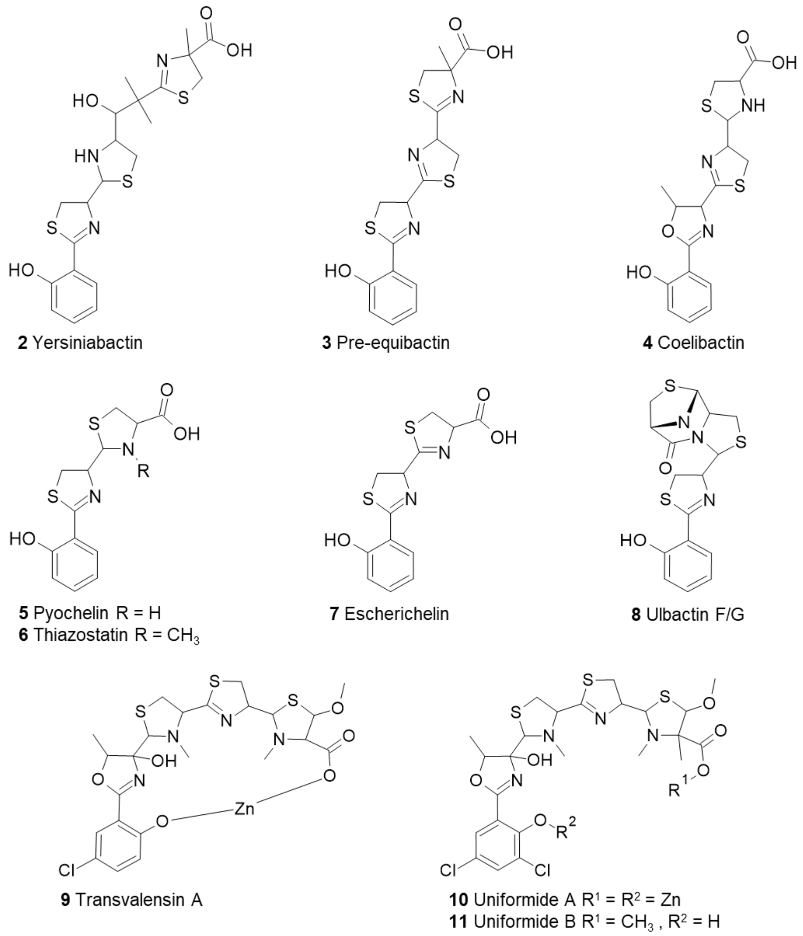

Supplementary Figure 3. Metabolites bearing structural similarities with the structure prediction of mupirochelin. Yersiniabactin (**2**), pyochelin (**5**), thiazostatin A (**6**), escherichelin (**7**) and ulbactin F/G (**8**), transvalencin A (**9**), uniformide A (**10**) and B (**11**) structures have been solved. Pre-equibactin (**3**) and coelibactin (**4**) structures are putative. Compounds **2**, **5**, **6**, **7**, **8** are arylthiazoline siderophores. Compounds **3** and **4** are aryloxazoline siderophores. Compounds **9**, **10**, and **11** are bioactive metabolites.

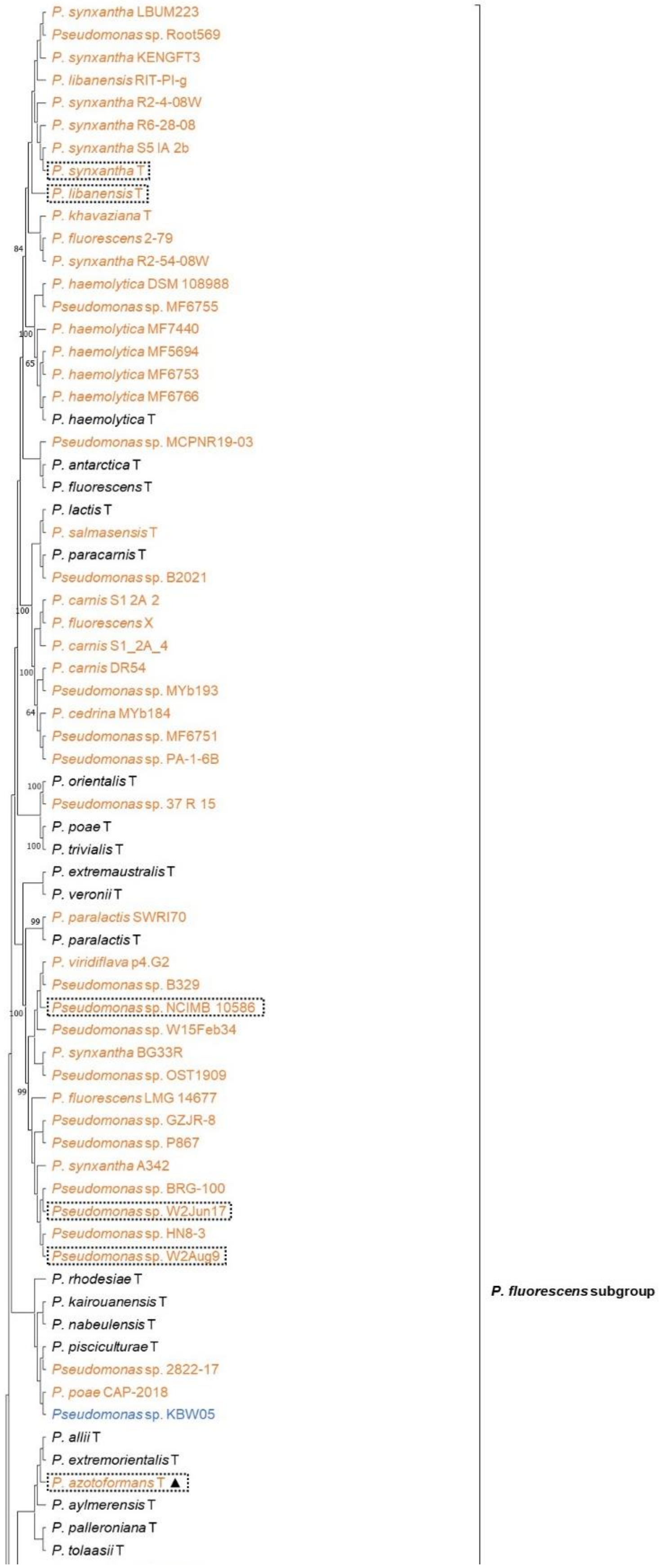
P. synxantha LBUM223
Pseudomonas sp. Root569
P. synxantha KENGFT3
P. libanensis RIT-PI-g
P. synxantha R2-4-08W
P. synxantha R6-28-08
P. synxantha S5 IA 2b
P. synxantha T
P. libanensis T
P. khavaziana T
P. fluorescens 2-79
P. synxantha R2-54-08W
P. haemolytica DSM 108988
Pseudomonas sp. MF6755
P. haemolytica MF7440
P. haemolytica MF5694
P. haemolytica MF6753
P. haemolytica MF6766
P. haemolytica T
Pseudomonas sp. MCPNR19-03
P. antarctica T
P. fluorescens T
P. lactis T
P. salmasensis T
P. paracarnis T
Pseudomonas sp. B2021
P. carnis S1 2A 2
P. fluorescens X
P. carnis S1_2A_4
P. carnis DR54
Pseudomonas sp. MYb193
P. cedrina MYb184
Pseudomonas sp. MF6751
Pseudomonas sp. PA-1-6B
P. orientalis T
Pseudomonas sp. 37 R 15
P. poae T
P. trivialis T
P. extremaustralis T
P. veronii T
P. paralactis SWRI70
P. paralactis T
P. viridiflava p4.G2
Pseudomonas sp. B329
Pseudomonas sp. NCIMB 10586
Pseudomonas sp. W15Feb34
P. synxantha BG33R
Pseudomonas sp. OST1909
P. fluorescens LMG 14677
Pseudomonas sp. GZJR-8
Pseudomonas sp. P867
P. synxantha A342
Pseudomonas sp. BRG-100
Pseudomonas sp. W2Jun17
Pseudomonas sp. HN8-3
Pseudomonas sp. W2Aug9
P. rhodesiae T
P. kairouanensis T
P. nabeulensis T
P. pisciculturae T
Pseudomonas sp. 2822-17
P. poae CAP-2018
Pseudomonas sp. KBW05
P. allii T
P. extremorientalis T
P. azotoformans T ▲
P. aylmerensis T
P. palleroniana T
P. tolaasii T
P. fluorescens subgroup

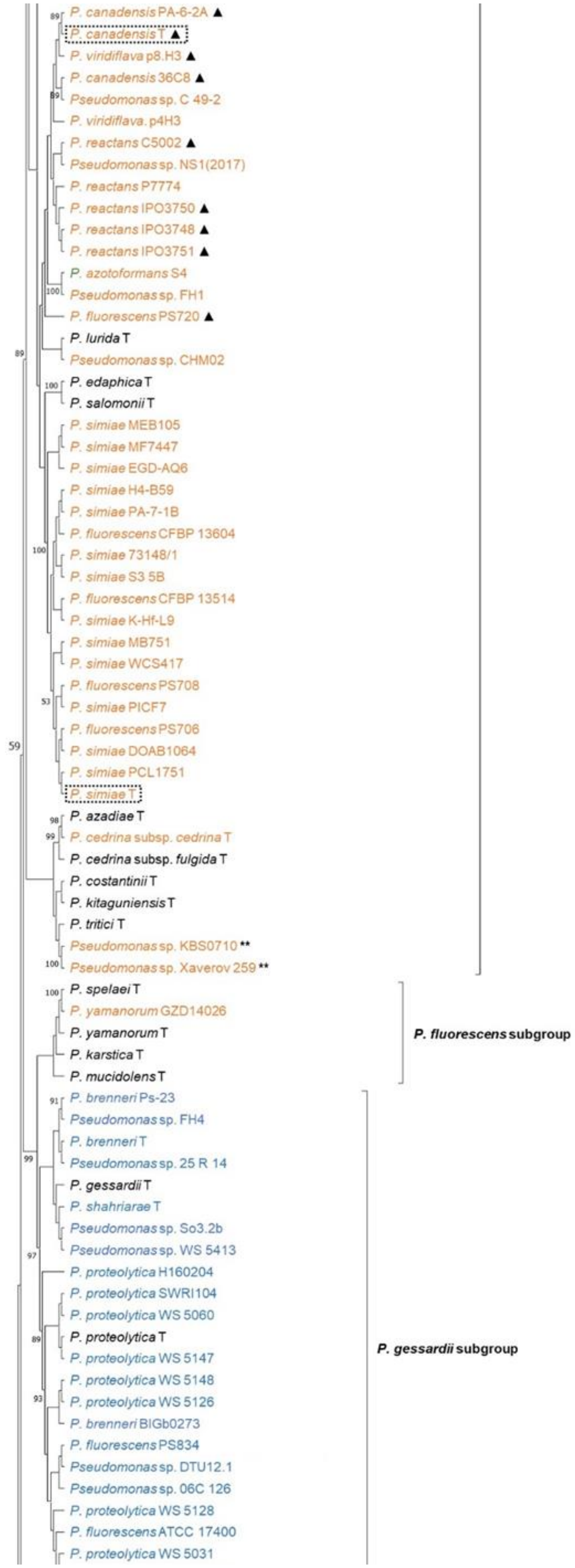
P. canadensis PA-6-2A ▲
P. canadensis T ▲
P. viridiflava p8.H3 ▲
P. canadensis 36C8 ▲
Pseudomonas sp. C 49-2
P. viridiflava. p4H3
P. reactans C5002 ▲
Pseudomonas sp. NS1(2017)
P. reactans P7774
P. reactans IPO3750 ▲
P. reactans IPO3748 ▲
P. reactans IPO3751 ▲
P. azotoformans S4
Pseudomonas sp. FH1
P. fluorescens PS720 ▲
P. lurida T
Pseudomonas sp. CHM02
P. edaphica T
P. salomonii T
P. simiae MEB105
P. simiae MF7447
P. simiae EGD-AQ6
P. simiae H4-B59
P. simiae PA-7-1B
P. fluorescens CFBP 13604
P. simiae 73148/1
P. simiae S3 5B
P. fluorescens CFBP 13514
P. simiae K-Hf-L9
P. simiae MB751
P. simiae WCS417
P. fluorescens PS708
P. simiae PICF7
P. fluorescens PS706
P. simiae DOAB1064
P. simiae PCL1751
P. simiae T
P. azadiae T
P. cedrina subsp. cedrina T
P. cedrina subsp. fulgida T
P. costantinii T
P. kitaguniensis T
P. tritici T
Pseudomonas sp. KBS0710 **
Pseudomonas sp. Xaverov 259 **
P. spelaei T
P. yamanorum GZD14026
P. yamanorum T
P. karstica T
P. mucidolens T
P. fluorescens subgroup
P. brenneri Ps-23
Pseudomonas sp. FH4
P. brenneri T
Pseudomonas sp. 25 R 14
P. gessardii T
P. shahriarae T
Pseudomonas sp. So3.2b
Pseudomonas sp. WS 5413
P. proteolytica H160204
P. proteolytica SWRI104
P. proteolytica WS 5060
P. proteolytica T
P. proteolytica WS 5147
P. proteolytica WS 5148
P. proteolytica WS 5126
P. brenneri BIGb0273
P. fluorescens PS834
Pseudomonas sp. DTU12.1
Pseudomonas sp. 06C 126
P. proteolytica WS 5128
P. fluorescens ATCC 17400
P. proteolytica WS 5031
P. gessardii subgroup
89
89
100
89
100
100
53
59
98
99
100
100
91
99
97
89
93

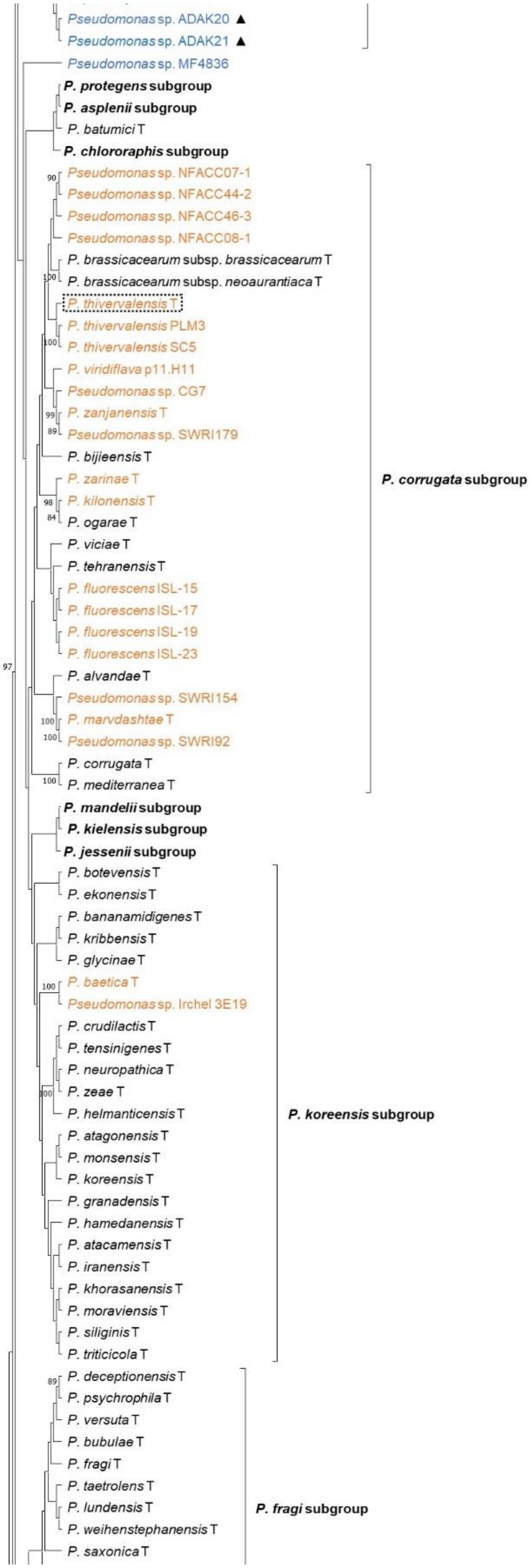

Pseudomonas sp. ADAK20 ▲
Pseudomonas sp. ADAK21 ▲
Pseudomonas sp. MF4836
P. protegens subgroup
P. asplenii subgroup
P. batumici T
P. chlororaphis subgroup
Pseudomonas sp. NFACC07-1
Pseudomonas sp. NFACC44-2
Pseudomonas sp. NFACC46-3
Pseudomonas sp. NFACC08-1
P. brassicacearum subsp. brassicacearum T
P. brassicacearum subsp. neoaurantiaca T
P. thivervalensis T
P. thivervalensis PLM3
P. thivervalensis SC5
P. viridiflava p11.H11
Pseudomonas sp. CG7
P. zanjanensis T
Pseudomonas sp. SWRI179
P. bijieensis T
P. zarinae T
P. kilonensis T
P. ogarae T
P. viciae T
P. tehranensis T
P. fluorescens ISL-15
P. fluorescens ISL-17
P. fluorescens ISL-19
P. fluorescens ISL-23
P. alvandae T
Pseudomonas sp. SWRI154
P. marvdashtae T
Pseudomonas sp. SWRI92
P. corrugata T
P. mediterranea T
P. corrugata subgroup
P. mandelii subgroup
P. kielensis subgroup
P. jessenii subgroup
P. botevensis T
P. ekonensis T
P. bananamidigenes T
P. kribbensis T
P. glycinae T
P. baetica T
Pseudomonas sp. Irchel 3E19
P. crudilactis T
P. tensinigenes T
P. neuropathica T
P. zeae T
P. helmanticensis T
P. atagonensis T
P. monsensis T
P. koreensis T
P. granadensis T
P. hamedanensis T
P. atacamensis T
P. iranensis T
P. khorasanensis T
P. moraviensis T
P. siliginis T
P. triticicola T
P. koreensis subgroup
P. deceptionensis T
P. psychrophila T
P. versuta T
P. bubulae T
P. fragi T
P. taetrolens T
P. lundensis T
P. weihenstephanensis T
P. saxonica T
P. fragi subgroup
90
100
100
99
89
98
84
97
100
100
100
100
100
89

frontiers

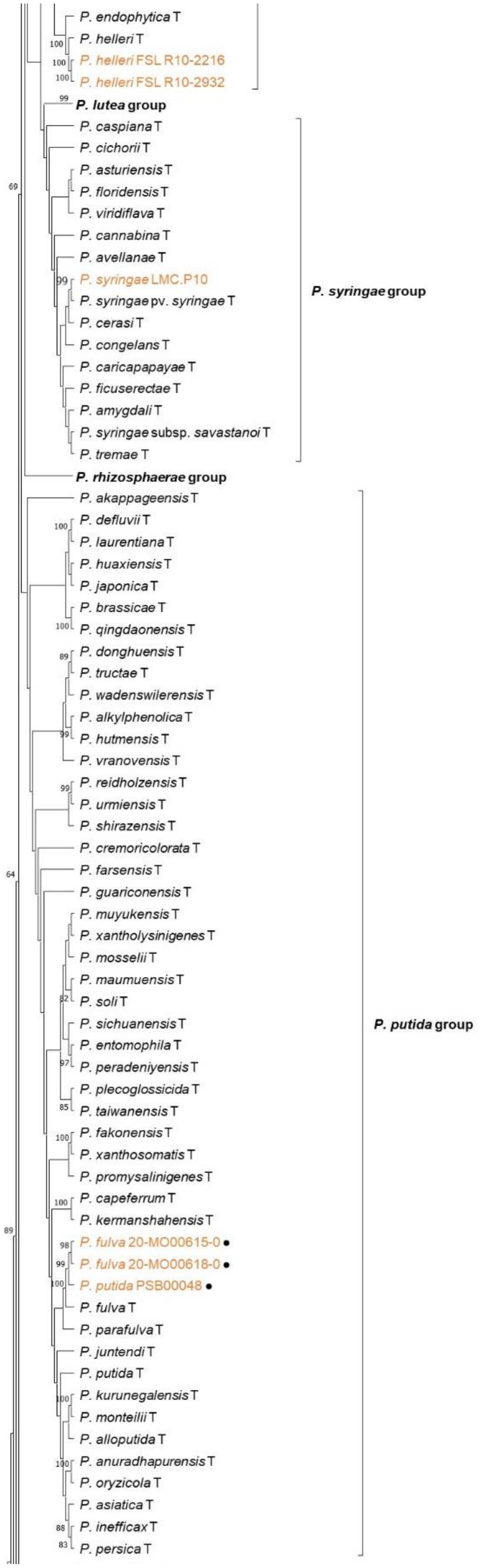
P. endophytica T
P. helleri T
P. helleri FSL R10-2216
P. helleri FSL R10-2932
P. lutea group
P. caspiana T
P. cichorii T
P. asturiensis T
P. floridensis T
P. viridiflava T
P. cannabina T
P. avellanae T
P. syringae LMC.P10
P. syringae pv. syringae T
P. cerasi T
P. congelans T
P. caricapapayae T
P. ficuserectae T
P. amygdali T
P. syringae subsp. savastanoi T
P. tremae T
P. syringae group
P. rhizosphaerae group
P. akappageensis T
P. defluvii T
P. laurentiana T
P. huaxiensis T
P. japonica T
P. brassicae T
P. qingdaonensis T
P. donghuensis T
P. tructae T
P. wadenswilerensis T
P. alkylphenolica T
P. hutmensis T
P. vranovensis T
P. reidholzensis T
P. urmiensis T
P. shirazensis T
P. cremoricolorata T
P. farsensis T
P. guariconensis T
P. muyukensis T
P. xantholysinigenes T
P. mosselii T
P. maumuensis T
P. soli T
P. sichuanensis T
P. entomophila T
P. peradeniyensis T
P. plecoglossicida T
P. taiwanensis T
P. fakonensis T
P. xanthosomatis T
P. promysalinigenes T
P. capeferrum T
P. kermanshahensis T
P. fulva 20-MO00615-0
P. fulva 20-MO00618-0
P. putida PSB00048
P. fulva T
P. parafulva T
P. juntendi T
P. putida T
P. kurunegalensis T
P. monteilii T
P. alloputida T
P. anuradhapurensis T
P. oryzicola T
P. asiatica T
P. inefficax T
P. persica T
P. putida group

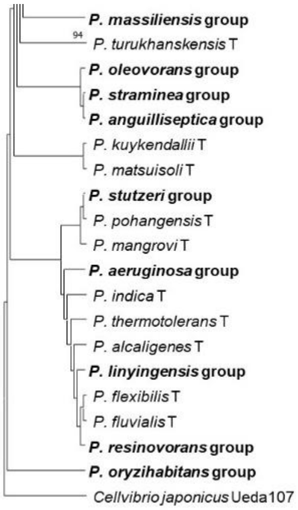

Supplementary Figure 4. Maximum likelihood tree. Maximum likelihood tree (TN + G+I model) based on partial *rpoD* gene sequences showing the *Pseudomonas* type strains and the position of strains predicted to produce mupirochelin (in orange) or to have only the mupirochelin transport and regulatory genes *mchMN*, *mchOPQ*, *mchS and mchR* (in blue). *Pseudomonas* strains that miss the isochorismate-pyruvate lyase gene are indicated with **. Strains that have a transposase between the *mchQ* and *mchS* genes are indicated with a ▲. The strains for which mupirochelin production has been determined by LC-MS are surrounded by a dotted line. The groups and subgroups are indicated in bold. Sub/groups that do not contain any candidate were compressed to reduce the size of the tree. *Cellvibrio japonicus* Ueda107 was used as outgroup. The bar indicates sequence divergence. Bootstrap values are indicated at branch points.

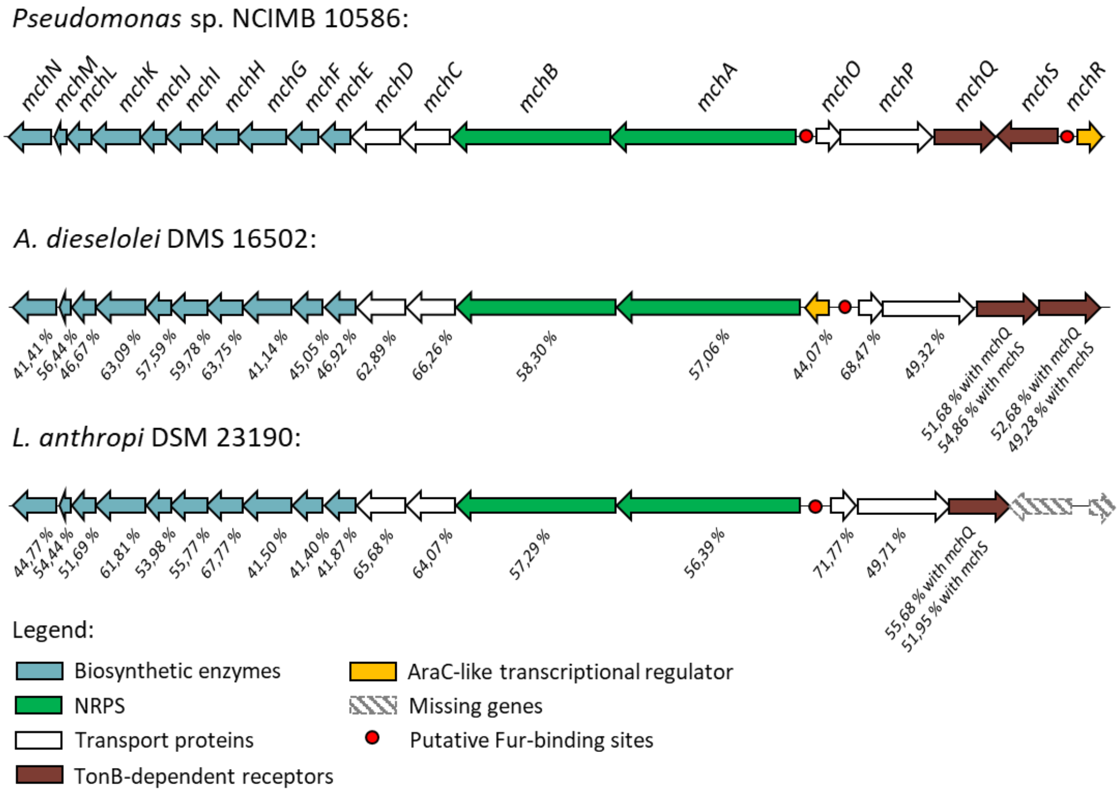

Supplementary Figure 5. Organization of the putative mupirochelin gene clusters found in *A. dieselolei* DMS 16502 and *L. anthropi* DSM 23190. Transposon insertions are indicated by black triangles. Red circles represent hypothetical Fur-binding sites. Genes missing in comparison with the *Pseudomonas* gene cluster are indicated by grey stripes. Percentages of amino acid identity with *Pseudomonas* sp. NCIMB 10586 genes are indicated under each ORF. *A. dieselolei* DSM 16502 gene cluster has a slightly different organization for the AraC-like transcriptional regulator and the two TonB-dependent receptors. In *L. anthropi* DSM 23190 the gene cluster is smaller due to the absence of the last TonB-dependent receptor and the AraC-like transcriptional regulator.

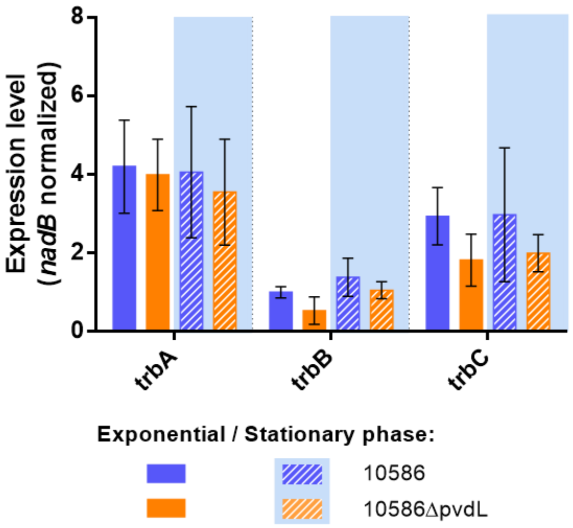

Supplementary Figure 6. Influence of pyoverdine production on the *trb* gene cluster expression. The gene cluster expression is not influenced by the absence of pyoverdine biosynthesis (n = 4). For a given gene, comparisons between groups were performed by Kruskal–Wallis test followed by Dunn's multiple comparisons test **p* < 0.05, ***p* <0.01, ****p* <0.001.
